# Clinical phenotypes and outcomes in children with multisystem inflammatory syndrome across SARS-CoV-2 variant eras: a multinational study from the 4CE consortium

**DOI:** 10.1016/j.eclinm.2023.102212

**Published:** 2023-09-14

**Authors:** Francesca Sperotto, Alba Gutiérrez-Sacristán, Simran Makwana, Xiudi Li, Valerie N. Rofeberg, Tianxi Cai, Florence T. Bourgeois, Gilbert S. Omenn, David A. Hanauer, Carlos Sáez, Clara-Lea Bonzel, Emily Bucholz, Audrey Dionne, Matthew D. Elias, Noelia García-Barrio, Tomás González González, Richard W. Issitt, Kate F. Kernan, Jessica Laird-Gion, Sarah E. Maidlow, Kenneth D. Mandl, Taha Mohseni Ahooyi, Cinta Moraleda, Michele Morris, Karyn L. Moshal, Miguel Pedrera-Jiménez, Mohsin A. Shah, Andrew M. South, Anastasia Spiridou, Deanne M. Taylor, Guillaume Verdy, Shyam Visweswaran, Xuan Wang, Zongqi Xia, Joany M. Zachariasse, The Consortium for Clinical Characterization of COVID-19 by EHR (4CE), James R. Aaron, Atif Adam, Giuseppe Agapito, Adem Albayrak, Giuseppe Albi, Mario Alessiani, Anna Alloni, Danilo F. Amendola, François Angoulvant, Li LLJ. Anthony, Bruce J. Aronow, Fatima Ashraf, Andrew Atz, Paul Avillach, Vidul Ayakulangara Panickan, Paula S. Azevedo, Rafael Badenes, James Balshi, Ashley Batugo, Brendin R. Beaulieu-Jones, Brett K. Beaulieu-Jones, Douglas S. Bell, Antonio Bellasi, Riccardo Bellazzi, Vincent Benoit, Michele Beraghi, José Luis Bernal-Sobrino, Mélodie Bernaux, Romain Bey, Surbhi Bhatnagar, Alvar Blanco-Martínez, Martin Boeker, Clara-Lea Bonzel, John Booth, Silvano Bosari, Florence T. Bourgeois, Robert L. Bradford, Gabriel A. Brat, Stéphane Bréant, Nicholas W. Brown, Raffaele Bruno, William A. Bryant, Mauro Bucalo, Emily Bucholz, Anita Burgun, Tianxi Cai, Mario Cannataro, Aldo Carmona, Anna Maria Cattelan, Charlotte Caucheteux, Julien Champ, Jin Chen, Krista Y. Chen, Luca Chiovato, Lorenzo Chiudinelli, Kelly Cho, James J. Cimino, Tiago K. Colicchio, Sylvie Cormont, Sébastien Cossin, Jean B. Craig, Juan Luis Cruz-Bermúdez, Jaime Cruz-Rojo, Arianna Dagliati, Mohamad Daniar, Christel Daniel, Priyam Das, Batsal Devkota, Audrey Dionne, Rui Duan, Julien Dubiel, Scott L. DuVall, Loic Esteve, Hossein Estiri, Shirley Fan, Robert W. Follett, Thomas Ganslandt, Noelia García-Barrio, Lana X. Garmire, Nils Gehlenborg, Emily J. Getzen, Alon Geva, Rachel SJ. Goh, Tomás González González, Tobias Gradinger, Alexandre Gramfort, Romain Griffier, Nicolas Griffon, Olivier Grisel, Alba Gutiérrez-Sacristán, Pietro H. Guzzi, Larry Han, David A. Hanauer, Christian Haverkamp, Derek Y. Hazard, Bing He, Darren W. Henderson, Martin Hilka, Yuk-Lam Ho, John H. Holmes, Jacqueline P. Honerlaw, Chuan Hong, Kenneth M. Huling, Meghan R. Hutch, Richard W. Issitt, Anne Sophie Jannot, Vianney Jouhet, Mundeep K. Kainth, Kernan F. Kate, Ramakanth Kavuluru, Mark S. Keller, Chris J. Kennedy, Kate F. Kernan, Daniel A. Key, Katie Kirchoff, Jeffrey G. Klann, Isaac S. Kohane, Ian D. Krantz, Detlef Kraska, Ashok K. Krishnamurthy, Sehi L'Yi, Judith Leblanc, Guillaume Lemaitre, Leslie Lenert, Damien Leprovost, Molei Liu, Ne Hooi Will Loh, Qi Long, Sara Lozano-Zahonero, Yuan Luo, Kristine E. Lynch, Sadiqa Mahmood, Sarah E. Maidlow, Adeline Makoudjou, Simran Makwana, Alberto Malovini, Kenneth D. Mandl, Chengsheng Mao, Anupama Maram, Monika Maripuri, Patricia Martel, Marcelo R. Martins, Jayson S. Marwaha, Aaron J. Masino, Maria Mazzitelli, Diego R. Mazzotti, Arthur Mensch, Marianna Milano, Marcos F. Minicucci, Bertrand Moal, Taha Mohseni Ahooyi, Jason H. Moore, Cinta Moraleda, Jeffrey S. Morris, Michele Morris, Karyn L. Moshal, Sajad Mousavi, Danielle L. Mowery, Douglas A. Murad, Shawn N. Murphy, Thomas P. Naughton, Carlos Tadeu Breda Neto, Antoine Neuraz, Jane Newburger, Kee Yuan Ngiam, Wanjiku FM. Njoroge, James B. Norman, Jihad Obeid, Marina P. Okoshi, Karen L. Olson, Gilbert S. Omenn, Nina Orlova, Brian D. Ostasiewski, Nathan P. Palmer, Nicolas Paris, Lav P. Patel, Miguel Pedrera-Jiménez, Ashley C. Pfaff, Emily R. Pfaff, Danielle Pillion, Sara Pizzimenti, Tanu Priya, Hans U. Prokosch, Robson A. Prudente, Andrea Prunotto, Víctor Quirós-González, Rachel B. Ramoni, Maryna Raskin, Siegbert Rieg, Gustavo Roig-Domínguez, Pablo Rojo, Nekane Romero-Garcia, Paula Rubio-Mayo, Paolo Sacchi, Carlos Sáez, Elisa Salamanca, Malarkodi Jebathilagam Samayamuthu, L. Nelson Sanchez-Pinto, Arnaud Sandrin, Nandhini Santhanam, Janaina C.C. Santos, Fernando J. Sanz Vidorreta, Maria Savino, Emily R. Schriver, Petra Schubert, Juergen Schuettler, Luigia Scudeller, Neil J. Sebire, Pablo Serrano-Balazote, Patricia Serre, Arnaud Serret-Larmande, Mohsin A. Shah, Zahra Shakeri Hossein Abad, Domenick Silvio, Piotr Sliz, Jiyeon Son, Charles Sonday, Andrew M. South, Francesca Sperotto, Anastasia Spiridou, Zachary H. Strasser, Amelia LM. Tan, Bryce W.Q. Tan, Byorn W.L. Tan, Suzana E. Tanni, Deanne M. Taylor, Ana I. Terriza-Torres, Valentina Tibollo, Patric Tippmann, Emma MS. Toh, Carlo Torti, Enrico M. Trecarichi, Andrew K. Vallejos, Gael Varoquaux, Margaret E. Vella, Guillaume Verdy, Jill-Jênn Vie, Shyam Visweswaran, Michele Vitacca, Kavishwar B. Wagholikar, Lemuel R. Waitman, Xuan Wang, Demian Wassermann, Griffin M. Weber, Martin Wolkewitz, Scott Wong, Zongqi Xia, Xin Xiong, Ye Ye, Nadir Yehya, William Yuan, Joany M. Zachariasse, Janet J. Zahner, Alberto Zambelli, Harrison G. Zhang, Daniela Zöller, Valentina Zuccaro, Chiara Zucco, Jane W. Newburger, Paul Avillach

**Affiliations:** a Department of Cardiology, Boston Children's Hospital, Harvard Medical School, 300 Longwood Ave, Boston, MA 02115, United States; b Department of Biomedical Informatics, Harvard Medical School, 10 Shattuck Street, Boston, MA 02115, United States; c Department of Biostatistics, Harvard School of Public Health, 677 Huntington Ave, Boston, MA 02115, United States; d Department of Pediatrics, Harvard Medical School, 300 Longwood Ave, Boston, MA 02115, United States; e Dept of Computational Medicine & Bioinformatics, Internal Medicine, Human Genetics, & Public Health, University of Michigan, 2017 Palmer Commons, Ann Arbor, MI 48109-2218, United States; f Department of Learning Health Sciences, University of Michigan Medical School, 100-107 NCRC, 2800 Plymouth Road, Ann Arbor, MI 48109, United States; g Biomedical Data Science Lab, Instituto Universitario de Tecnologías de la Información y Comunicaciones, Universitat Politécnica de Valéncia, Camino de Vera S/N, Valencia 46022, Spain; h Department of Cardiology, Children's Hospital Colorado, University of Colorado Anschutz, 13123 E. 16th Ave, Aurora, CO 80045, United States; i Division of Cardiology, The Children's Hospital of Philadelphia, 3401 Civic Center Boulevard, Philadelphia, PA 19104, United States; j Health Informatics, Hospital Universitario 12 de Octubre, Av. de Córdoba, s/n, Madrid 28041, Spain; k Digital Research, Informatics and Virtual Environments (DRIVE), Great Ormond Street Hospital for Children, Great Ormond Street, London WC1N 3JH, United Kingdom; l Department of Critical Care Medicine, University of Pittsburgh, 3550 Terrace Street, Pittsburgh, PA 15213, United States; m Department of Pediatrics, Boston Children's Hospital, Harvard Medical School, 300 Longwood Ave, Boston, MA 02115, United States; n Michigan Institute for Clinical and Health Research (MICHR) Informatics, University of Michigan, NCRC Bldg 400, 2800 Plymouth Road, Ann Arbor, MI 48109, United States; o Computational Health Informatics Program, Boston Children's Hospital, 300 Longwood Avenue, Boston, MA 02115, United States; p Department of Biomedical Health Informatics, The Children's Hospital of Philadelphia, Roberts Building, 734 Schuylkill Ave, Philadelphia, PA 19146, United States; q Pediatric Infectious Disease Department, Hospital Universitario 12 de Octubre, Av. de Córdoba, s/n, Madrid 28041, Spain; r Department of Biomedical Informatics, University of Pittsburgh, 5607 Baum Blvd, Pittsburgh, PA 15206, United States; s Department of Infectious Diseases, Great Ormond Street Hospital for Children, Great Ormond Street, London WC1N 3JH, United Kingdom; t Digital Research, Informatics and Virtual Environments (DRIVE), Great Ormond Street Hospital for Children, DRIVE, 40 Bernard St, London WC1N 1LE, United Kingdom; u Department of Pediatrics-Section of Nephrology, Brenner Children’s, Wake Forest University School of Medicine, Medical Center Boulevard, Winston Salem, NC 27157, United States; v Data Research, Innovation and Virtual Environments, Great Ormond Street Hospital for Children, DRIVE, 40 Bernard St, London WC1N 1LE, United Kingdom; w Department of Biomedical Health Informatics, The Children's Hospital of Philadelphia, United States; x The Department of Pediatrics, University of Pennsylvania Perelman Medical School, 3601 Civic Center Blvd, 6032 Colket, Philadelphia, PA 19104, United States; y IAM Unit, Bordeaux University Hospital, Place amélie rabat Léon, Bordeaux 33076, France; z Department of Neurology, University of Pittsburgh, 3501 5th Avenue, BST-3 Suite 7014, Pittsburgh, PA 15260, United States

**Keywords:** Multisystem inflammatory syndrome, Paediatric inflammatory multisystem syndrome, COVID-19, SARS-CoV-2, Variants, Pediatrics, Clinical phenotypes, Outcomes

## Abstract

**Background:**

Multisystem inflammatory syndrome in children (MIS-C) is a severe complication of SARS-CoV-2 infection. It remains unclear how MIS-C phenotypes vary across SARS-CoV-2 variants. We aimed to investigate clinical characteristics and outcomes of MIS-C across SARS-CoV-2 eras.

**Methods:**

We performed a multicentre observational retrospective study including seven paediatric hospitals in four countries (France, Spain, U.K., and U.S.). All consecutive confirmed patients with MIS-C hospitalised between February 1st, 2020, and May 31st, 2022, were included. Electronic Health Records (EHR) data were used to calculate pooled risk differences (RD) and effect sizes (ES) at site level, using *Alpha* as reference. Meta-analysis was used to pool data across sites.

**Findings:**

Of 598 patients with MIS-C (61% male, 39% female; mean age 9.7 years [SD 4.5]), 383 (64%) were admitted in the *Alpha* era, 111 (19%) in the *Delta* era, and 104 (17%) in the *Omicron* era. Compared with patients admitted in the *Alpha* era, those admitted in the *Delta* era were younger (ES −1.18 years [95% CI −2.05, −0.32]), had fewer respiratory symptoms (RD −0.15 [95% CI −0.33, −0.04]), less frequent non-cardiogenic shock or systemic inflammatory response syndrome (SIRS) (RD −0.35 [95% CI −0.64, −0.07]), lower lymphocyte count (ES −0.16 × 10^9^/uL [95% CI −0.30, −0.01]), lower C-reactive protein (ES −28.5 mg/L [95% CI −46.3, −10.7]), and lower troponin (ES −0.14 ng/mL [95% CI −0.26, −0.03]). Patients admitted in the *Omicron* versus *Alpha* eras were younger (ES −1.6 years [95% CI −2.5, −0.8]), had less frequent SIRS (RD −0.18 [95% CI −0.30, −0.05]), lower lymphocyte count (ES −0.39 × 10^9^/uL [95% CI −0.52, −0.25]), lower troponin (ES −0.16 ng/mL [95% CI −0.30, −0.01]) and less frequently received anticoagulation therapy (RD −0.19 [95% CI −0.37, −0.04]). Length of hospitalization was shorter in the *Delta* versus *Alpha* eras (−1.3 days [95% CI −2.3, −0.4]).

**Interpretation:**

Our study suggested that MIS-C clinical phenotypes varied across SARS-CoV-2 eras, with patients in *Delta* and *Omicron* eras being younger and less sick. EHR data can be effectively leveraged to identify rare complications of pandemic diseases and their variation over time.

**Funding:**

None.

**Research in context**

**Evidence before this study**

Multisystem inflammatory syndrome in children (MIS-C), a post-infectious vasculitis associated with SARS-CoV-2 infection, represents one of the most important complications of COVID-19 in children and young adults. While outcomes after acute COVID-19 differ based on SARS-CoV-2 variants, data on variant-specific MIS-C phenotypes and outcomes are limited. To investigate the available evidence, we searched PubMed using the following search strategy, which included both *terms* and *controlled vocabulary terms*: “(COVID-19 OR SARS-CoV-2 OR COVID19 [MeSH Terms]) AND (“multisystem inflammatory syndrome” OR MISC OR MIS-C OR “multi-system inflammatory syndrome” OR “paediatric inflammatory multisystem syndrome” OR PIMS) AND (variant OR variants)” (last search date: May 15th, 2023). The search retrieved 130 documents; of them, eight addressed the research question. Most of the studies either had a small sample size or were monocentre. Preliminary results from a large register-based study suggested that patients hospitalised during the initial period of the pandemic were at higher risk of admission to the intensive care unit and present with ventricular dysfunction compared to those hospitalised during the most recent eras.

**Added value of this study**

Our multicentre multinational EHR-based study, which brings together data from a large cohort of patients with MIS-C from four countries across two continents, provided evidence suggesting that MIS-C clinical and laboratory characteristics and outcomes vary according to SARS-CoV-2 variant eras. We found that patients admitted during the *Delta* and *Omicron* eras were younger and less sick than those admitted in the *Alpha* era. Specifically, patients admitted during the *Alpha* era versus subsequent variant eras had more respiratory involvement and more frequently presented with shock or systemic inflammatory response syndrome (SIRS); they also had higher C-reactive protein, absolute lymphocyte count, and troponin levels; lower albumin; and longer hospitalization.

**Implications of all the available evidence**

We believe our study adds valuable information in characterizing different MIS-C phenotypes across SARS-CoV-2 eras and continents. This available evidence may help in risk stratification and clinical prognostication in paediatric patients with MIS-C. Our study also showed that EHR data may be effectively leveraged to investigate rare complications of diseases in the setting of the pandemic and used to identify patterns of disease variation or severity over time.

## Introduction

Multisystem inflammatory syndrome in children (MIS-C) is a post-infectious vasculitis associated with SARS-CoV-2 infection and represents one of the most important complications secondary to SARS-CoV-2 infection in children and young adults.^1^^,^^2^ Since the beginning of the pandemic, multiple SARS-CoV-2 variants, including *Alpha*, *Delta*, and *Omicron*,^3^ have been identified. Outcomes after acute COVID-19 in children have been reported to differ based on SARS-CoV-2 variants, with *Omicron* being associated with less severe illness than the *Alpha* and *Delta* variants.^4^ However, data on variant-specific MIS-C phenotypes and outcomes are still limited.

A report from the Centres for Disease Control and Prevention (CDC) — using U.S. voluntary national surveillance data from February 2020 to July 2021 — first observed that MIS-C clinical characteristics and outcomes appeared to vary across SARS-CoV-2 waves, with an overall decrease in the incidence of severe outcomes over time.^5^ This analysis was reproduced using data up to January 2022 and confirmed that MIS-C severity decreased over time.^6^ An analysis from Israel of data from 171 patients admitted in 12 centres showed that cardiovascular outcomes were more favourable during the *Omicron* wave and length of hospitalization was shorter compared to previous waves.^7^ A recent large cohort study from the International Kawasaki Disease Registry demonstrated that, compared to patients hospitalised during the ancestral period (pre-*Alpha*), the risk of intensive care unit (ICU) admission was lowest in those hospitalised during the *Omicron* era, and the risk of ventricular dysfunction was highest among those hospitalised during the *Alpha* era.^8^ However, other smaller single-centre and multicentre reports from South Africa, Europe, and the U.S. found no difference in MIS-C outcomes across SAS-CoV-2 variant eras.^9^, ^10^, ^11^, ^12^, ^13^

The Consortium for Clinical Characterization of COVID-19 (4CE) is an international consortium that brings together researchers and electronic health record (EHR) data scientists to leverage EHR data using a federated approach to address research questions related to COVID-19, while preserving data confidentiality.^14^ We aimed to investigate and compare clinical characteristics, laboratory data, and patient-level outcomes of patients with MIS-C who were hospitalised during different SARS-CoV-2 eras using multicentre data from the 4CE Consortium.

## Methods

### Study design, setting, and population

**Fig. 1:**
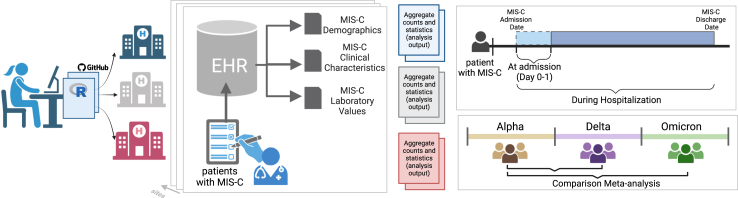
Graphical summary of the study methodological structure. MIS-C: multisystem inflammatory syndrome.

We performed a retrospective multicentre population-based study using 4CE Consortium data from 4 countries (France, Spain, U.K., and U.S.; Supplemental Table S1). The overall study methodological structure is summarized in Fig. 1. All analyses involving identifiable data were conducted at the site level; aggregate analyses were performed at the project coordinating centre (Boston Children’s Hospital, BCH). A list of consecutive patients with MIS-C was identified by MIS-C experts at each site. Inclusion criteria were age <21 years, diagnosis of MIS-C based on CDC, Royal College of Paediatrics and Child Health, or World Health Organization criteria according to institutional practice, and hospitalization for MIS-C between February 1st, 2020 and May 31st, 2022 (Supplemental Tables S1 and S2). Approval for the study was obtained from the institutional review board at each site with a waiver of informed consent since only de-identified retrospective observational data were analysed. The study was conducted following the ethical principles for medical research of the Helsinki Declaration and the quality standard required by the Strengthening the Reporting of Observational Studies in Epidemiology (STROBE) guidelines.

### Data extraction, quality check, and data definition

Data were collected from the EHR of participating sites in a federated approach (Supplemental Fig. S1). Patient-level data at each site were extracted following the 4CE common data model. An R package was created for site-level analyses and shared with all sites following a step-by-step approach (Supplemental Fig. S1). Aggregated counts and statistics were then shared centrally. The code is publicly available on GitHub (GitHub Inc., San Francisco, U.S. https://github.com/covidclinical/PhaseX.2SqlDataExtraction/tree/main/Extras, https://github.com/covidclinical/Phase2.2MISCRPackage).

Data quality checks were performed at site level and across sites (Supplemental Methods). To facilitate this process, a data visualization tool was developed to compare International Classification of Disease 10th revision (ICD-10) codes, counts, laboratory values, and summary statistics across sites (R Shiny App, R Foundation for Statistical Computing, Vienna, Austria), which is publicly available (https://avillachlab.shinyapps.io/a1l1non3/). Two sites applied obfuscation thresholds for small counts to minimize disclosure risks related to small numbers of patients (Supplemental Table S1). When count values were obfuscated, a value of 1 was adopted.

Data included age, sex, clinical characteristics, laboratory data, and patient-level outcomes during the hospitalization of interest. Clinical characteristics were based on EHR-extracted ICD-10 codes. A detailed list of clinical characteristics based on ICD-10 codes is reported in the Supplemental Methods. Of note, one centre did not pass the quality check control for ICD-10 code-based data; therefore, ICD-10 code-based data of this centre were excluded from the analysis. Laboratory data at admission (day 0–1) and the *worst* value during hospitalization were extracted from the EHR. Definitions of *worst* value for each laboratory data is reported in Supplemental Table S8. Patient-level outcomes — used as a measure of severity — included ICU admission, oxygen supplementation or mechanical ventilation (MV), diuretic therapy, anticoagulation therapy, vasoactive/inotropic support, use of sedation/muscle-relaxants, cannulation to extracorporeal membrane oxygenation (ECMO), cardiac arrest, length of hospitalization, and in-hospital mortality. A composite adverse cardiovascular outcome measure was also investigated, defined as presence of at least one among ventricular dysfunction, heart failure or cardiogenic shock, inotropic/vasoactive drugs, coronary aneurysm, major arrhythmias, cardiac arrest, or veno-arterial ECMO. Definitions of outcomes based on ICD-10 codes or EHR-mapped elements is reported in the Supplemental Methods.

### Definition of SARS-CoV-2 variant eras

The Global Initiative on Sharing All Influenza Data (GISAID) — an initiative developed for collecting epidemiologic data on influenza viruses that expanded its expertise on SARS-CoV-2 data during the pandemic — was used to identify SARS-CoV-2 variant eras based on variant predominance by country.^15^ For European sites, all cases hospitalised up to April 30, 2021 were assigned to *Alpha*, up to December 31, 2021 to *Delta*, and up to the end of the study, May 31, 2022, to *Omicron*. For U.S. sites, all cases hospitalised up to June 30, 2021, were assigned to *Alpha*, up to December 31, 2021, to *Delta*, and up to May 31, 2022, to *Omicron*. Since MIS-C may have delayed onset compared to SARS-CoV-2 infection.^1^^,^^16^ we conducted a sensitivity analysis by shifting the cut-off dates to two weeks later.

### Statistical analysis

Data were summarized as counts and percentages for categorical variables, and means and standard deviations (SDs) for continuous variables at the site level. At the federated level, for descriptive purposes, these summary data were used to compute aggregate counts and percentages for categorical variables, as well as pooled means and pooled SDs for continuous variables. To compare patients’ characteristics and outcomes across the SARS-CoV-2 variant eras, we used meta-analysis methods based on the variable type. All comparisons were made using the *Alpha* era as reference. For categorical variables, we computed risk differences (RDs) with 95% confidence intervals (CIs) at the site level, and subsequently pooled RDs with 95% CIs across sites. To calculate the pooled RDs and 95% CIs, we employed a meta-analysis method specifically developed for small sample sizes.^17^ Unlike the conventional meta-analysis procedures, this method provides valid exact inferences under a fixed-effects framework effectively utilizing all data while not relying on the large-sample approximation or arbitrary continuity corrections.^17^ For continuous variables, we estimated the effect sizes (ES) using the difference in means between groups. We first computed the ES and 95% CI at the site level and subsequently pooled these across sites using fixed-effects meta-analysis. The same analyses were reproduced, as a sensitivity analysis, with the era cut-off dates shifted two weeks later. All statistical analyses were performed using R statistics (version 3.6.2., R Core Team, R Foundation for Statistical Computing, Vienna, Austria).

### Role of the funding source

All authors had full access to all the data, accept full responsibility of ensuring accuracy or integrity of any part of the work, approved the final version of the manuscript and agreed to submit it for publication. There was no funding source for this study.

## Results

**Fig. 2:**
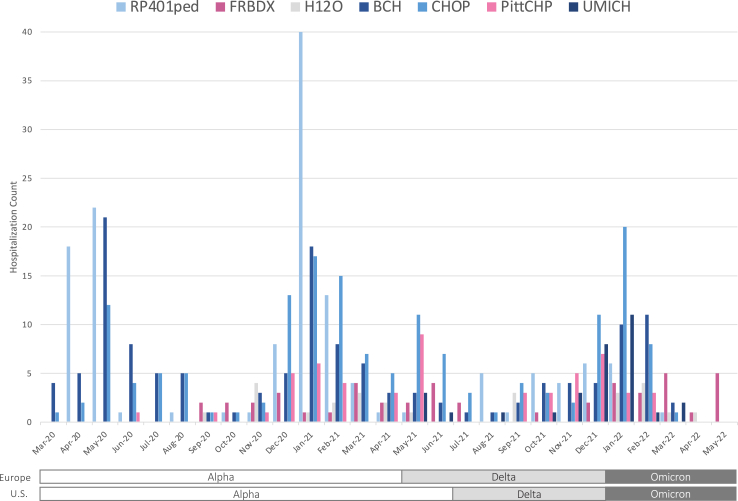
Distribution of MIS-C count over time in relation to SARS-CoV-2 variant eras. Centres are showed by longitude starting from east to west, and are indicated by colours and official abbreviations as follows: RP401ped: Great Ormond Street Hospital for Children, London, U.K.; FRBDX: Bordeaux University Hospital, Bordeaux, France; H12O: Hospital Universitario 12 de Octubre, Madrid, Spain; BCH: Boston Children’s Hospital, Boston, U.S.; CHOP: The Children’s Hospital of Philadelphia, Philadelphia, U.S.; PittCHP: University of Pittsburgh Medical Center, Pittsburgh, U.S.; UMICH: University of Michigan, U.S.

Among 598 MIS-C who were hospitalised in 7 participating sites (61% male, 39% female; pooled mean age 9.7 years [pooled SD 4.5]), 383 (64%) were admitted in the *Alpha* era, 111 (19%) in the *Delta* era, and 104 (17%) in the *Omicron* era. The distribution of MIS-C count over time in relation to SARS-CoV-2 variant eras is shown in Fig. 2.

### Demographic and clinical characteristics

Patients’ demographic and clinical characteristics for the whole cohort and across SARS-CoV-2 eras are shown in Table 1 and Supplemental Table S3. Overall, the most common clinical manifestations were cardiovascular involvement (74%), followed by respiratory involvement (51%), and neurologic involvement (37%). Conjunctivitis was reported in 35% and a rash is 33%. A systemic inflammatory response syndrome (SIRS) was reported in 24%.

**Table 1:** Demographic and clinical characteristics of patients with MIS-C according to SARS-CoV-2 variant era.

Variable	Total, N = 436^a^	MIS-C during A*lpha* era, N = 275	MIS-C during *Delta* era, N = 87	MIS-C during O*micron* era, N = 74	*Delta* compared to A*lpha*		*Omicron* compared to A*lpha*	
					Pooled RD or ES (95% CI)	P value	Pooled RD or ES (95% CI)	P value
Age, years, pooled mean (pooled SD) [N]	9.7 (4.5) [N = 598]	10.3 (4.8) [N = 383]	9.0 (3.9) [N = 111]	8.2 (3.8) [N = 104]	−1.183 (−2.049, −0.317)	**0.007**	−1.649 (−2.535, −0.763)	**<0.001**
Sex, N (%)								
Male	365 (61.0)	231 (60.3)	68 (61.3)	66 (63.5)	0.012 (−0.097, −0.118)^b^	0.884	0.045 (−0.178, 0.195)^b^	1.000
Female	233 (39.0), [N = 598]	152 (39.7) [N = 383]	43 (38.7) [N = 111]	38 (36.5) [N = 104]				
**Baseline comorbidities**								
Overweight or obesity, N (%)	28 (6.4)	23 (8.4)	2 (2.2)	3 (4.0)	−0.065 (−0.131, 0.021)	0.176	−0.088 (−0.145, 0.047)	0.229
Asthma, N (%)	36 (8.3)	23 (8.4)	5 (5.7)	8 (10.8)	−0.036 (−0.120, 0.042)	0.573	0.032 (−0.087, 0.146)	1.000
**Generalized symptoms** (other than fever) **or muco-cutaneous involvement**, N (%)	310 (71.1)	201 (73.1)	58 (66.7)	51 (68.9)	−0.013 (−0.213, 0.102)	1.000	−0.047 (−0.265, 0.156)	1.000
Fatigue, asthenia	91 (20.9)	62 (22.5)	15 (17.2)	14 (18.9)	−0.035 (−0.124, 0.113)	0.799	−0.000 (−0.193, 0.128)	1.000
Rash	143 (32.8)	95 (34.5)	24 (27.5)	24 (32.4)	0.036 (−0.153, 0.166)	0.935	−0.002 (−0.124, 0.124)	1.000
Conjunctivitis	155 (35.5)	99 (36.0)	31 (35.6)	25 (33.8)	0.118 (−0.072, 0.234)	0.184	0.008 (−0.122, 0.166)	1.000
Mucositis	52 (11.9)	38 (13.8)	7 (8.1)	7 (9.5)	−0.008 (−0.126, 0.071)	0.961	−0.057 (−0.120, 0.032)	0.174
Lymphadenitis/lymphadenopathy	72 (16.5)	47 (17.1)	11 (12.6)	14 (18.9)	−0.072 (−0.210, 0.057)	0.986	0.000 (−0.129, 0.120)	1.000
**Gastrointestinal involvement**, N (%)	248 (56.9)	165 (60.0)	49 (56.3)	34 (45.9)	0.069 (−0.133, 0.265)	0.647	0.049 (−0.341, 0.273)	1.000
Abdominal pain	148 (33.9)	101 (36.7)	30 (34.5)	17 (23.0)	0.018 (−0.143, 0.232)	1.000	−0.033 (−0.256, 0.184)	1.000
Nausea or vomiting	97 (22.2)	65 (23.6)	18 (20.7)	14 (18.9)	−0.011 (−0.170, 0.161)	1.000	−0.015 (−0.252, 0.243)	1.000
Diarrhea, enteritis, ileitis	101 (23.2)	75 (27.3)	15 (17.2)	11 (14.9)	−0.077 (−0.194, 0.137)	0.687	−0.014 (−0.186, 0.110)	1.000
Appendicitis, peritonitis	3 (0.7)	1 (0.4)	0 (0.0)	2 (2.7)	−0.001 (−0.030, 0.044)	1.000	0.032 (−0.018, 0.111)	0.659
**Respiratory involvement**, N (%)	222 (50.9)	162 (58.9)	29 (33.3)	31 (41.9)	−0.148 (−0.327, −0.043)	**0.005**	−0.145 (−0.356, 0.054)	0.376
Cough	28 (6.4)	24 (8.7)	2 (2.3)	2 (2.7)	−0.037 (−0.096, 0.036)	0.300	−0.073 (−0.130, 0.024)	0.116
Rhinitis/Rhinorrhea	7 (1.6)	4 (1.4)	1 (1.1)	2 (2.7)	0.000 (−0.041, 0.067)	1.000	0.015 (−0.050, 0.089)	1.000
Sore throat	26 (6.0)	20 (7.3)	2 (2.3)	4 (5.4)	−0.035 (−0.085, 0.038)	0.513	0.000 (−0.093, 0.099)	1.000
Respiratory failure/dyspnea	47 (10.8)	30 (10.9)	9 (10.3)	8 (10.8)	−0.023 (−0.097, 0.067)	0.909	−0.035 (−0.121, 0.054)	0.924
Pleural effusion	81 (18.6)	59 (21.4)	11 (12.6)	11 (14.9)	−0.040 (−0.157, 0.056)	1.000	−0.021 (−0.181, 0.109)	1.000
Pulmonary edema	26 (6.0)	17 (6.2)	3 (3.4)	6 (8.1)	−0.001 (−0.052, 0.064)	1.000	0.051 (−0.030, 0.155)	0.258
Pneumonia	55 (12.6)	52 (18.9)	1 (1.1)	2 (2.7)	−0.129 (−0.258, −0.036)	**0.005**	−0.145 (−0.295, 0.033)	0.223
ARDS	9 (2.1)	6 (2.2)	1 (1.1)	2 (2.7)	0.002 (−0.043, 0.060)	1.000	−0.025 (−0.066, 0.045)	0.496
**Cardiovascular involvement**, N (%)	321 (73.6)	223 (81.1)	53 (60.9)	45 (60.8)	−0.043 (−0.332, 0.075)	0.825	−0.020 (−0.415, 0.130)	1.000
Chest pain	18 (4.1)	15 (5.4)	1 (1.1)	2 (2.7)	−0.020 (−0.072, 0.031)	0.511	−0.027 (−0.095, 0.051)	0.591
Hypotension	174 (39.9)	117 (42.5)	31 (35.6)	26 (35.1)	−0.029 (−0.146, 0.224)	1.000	0.003 (−0.160, 0.401)	1.000
Pre-syncope, syncope	54 (12.4)	36 (13.1)	13 (14.9)	5 (6.7)	0.068 (−0.012, 0.156)	0.234	−0.010 (−0.096, 0.080)	0.898
Arrhythmias	97 (22.2)	69 (25.1)	14 (16.1)	14 (18.9)	−0.003 (−0.075, 0.076)	1.000	−0.032 (−0.119, 0.064)	0.645
Myocarditis	58 (13.3)	41 (14.9)	7 (8.0)	10 (13.5)	−0.075 (−0.245, 0.024)	0.166	−0.059 (−0.298, 0.063)	0.585
Pericarditis/pericardial effusion	107 (24.5)	74 (26.9)	19 (21.8)	14 (18.9)	0.036 (−0.088, 0.138)	0.898	−0.014 (−0.146, 0.123)	1.000
Left ventricular dysfunction	60 (13.8)	43 (15.6)	6 (6.9)	11 (14.9)	−0.032 (−0.084, 0.031)	0.318	−0.012 (−0.103, 0.117)	1.000
Heart failure	56 (12.8)	41 (14.9)	10 (11.5)	5 (6.8)	−0.022 (−0.084, 0.089)	1.000	0.067 (−0.178, 0.043)	0.553
Cardiogenic shock	84 (19.3)	52 (18.9)	16 (18.4)	16 (21.6)	−0.076 (−0.169, 0.103)	0.652	0.017 (−0.201, 0.192)	1.000
**Shock (non-cardiogenic)/SIRS**, N (%)	187 (42.9)	146 (53.1)	16 (18.4)	23 (31.1)	−0.348 (−0.645, −0.067)	**0.006**	−0.101 (−0.288, 0.041)	0.207
Septic shock	45 (10.3)	36 (13.1)	5 (5.7)	4 (5.4)	−0.107 (−0.175, 0.021)	0.106	−0.011 (−0.110, 0.057)	1.000
Hypovolemic shock	8 (1.83)	4 (1.4)	2 (2.3)	2 (2.7)	−0.004 (−0.037, 0.060)	1.000	0.047 (−0.029, 0.129)	0.266
Shock, others (non-cardiogenic)	90 (20.6)	63 (22.9)	14 (16.1)	13 (17.6)	−0.135 (−0.281, 0.064)	0.342	−0.001 (−0.179, 0.175)	1.000
SIRS	104 (23.8)	93 (33.8)	3 (3.4)	8 (10.8)	−0.278 (−0.463, −0.024)	**0.010**	−0.181 (−0.304, −0.050)	**0.004**
**Neurologic involvement**, N (%)	163 (37.4)	130 (47.3)	19 (21.8)	14 (18.9)	−0.130 (−0.297, 0.026)	0.317	−0.048 (−0.371, 0.104)	0.958
Headache	54 (12.4)	43 (15.6)	4 (4.6)	7 (9.5)	−0.088 (−0.168, 0.002)	0.059	0.002 (−0.188, 0.117)	1.000
Disorientation/Confusion	27 (6.2)	23 (8.4)	2 (2.3)	2 (2.7)	−0.044 (0.120, 0.044)	0.437	−0.047 (−0.106, 0.044)	0.317
Seizures	9 (2.1)	6 (2.2)	2 (2.3)	1 (1.3)	0.013 (−0.037, 0.065)	0.948	−0.021 (−0.063, 0.042)	0.509
Muscle weakness/myalgia/myositis	47 (10.8)	35 (12.7)	9 (10.3)	3 (4.0)	0.031 (−0.070, 0.134)	0.935	0.021 (−0.146, 0.095)	1.000
Encephalopathy/meningoencephalitis	9 (2.1)	6 (2.2)	2 (2.3)	1 (1.3)	−0.047 (−0.186, 0.071)	0.841	−0.003 (−0.167, 0.048)	1.000
Stroke	3 (0.7)	2 (0.7)	0 (0)	1 (1.3)	0.000 (−0.032, 0.047)	1.000	0.017 (−0.039, 0.086)	0.749
**Kidney dysfunction**, N (%)	92 (21.1)	66 (24.0)	14 (16.1)	12 (16.2)	−0.048 (−0.233, 0.058)	0.540	0.000 (−0.273, 0.161)	1.000
**Liver dysfunction**, N (%)	50 (11.5)	32 (11.6)	7 (8.0)	11 (14.9)	−0.013 (−0.297, 0.026)	0.460	0.045 (−0.011, 0.217)	0.950

A detailed definition of the variables based on EHR data or ICD-10 codes is reported as Supplementary Material.

Aggregate counts and summary statistics for the total sample and the MIS-C era subgroups were calculated for descriptive purposes only. Meta-analyses were computed by pooling risk differences (RD, categorical variables) or effect sizes (ES, continuous variables) and their 95% confidence intervals (CIs) previously calculated at site-level. A detailed definition of the variables based on EHR data or ICD-10 codes is reported as Supplementary Material.

ES: effect size; MIS-C: multisystem inflammatory syndrome; RD: risk difference; SD: standard deviation; SIRS: systemic inflammatory response syndrome.

a ICD-10-code-based data from one centre, which did not pass the quality check, were excluded from analysis, thereby reducing sample size.

b Male as reference category.

Compared with patients admitted during the *Alpha* era, those admitted during the *Delta* era were younger (pooled ES −1.18 years [95% CI −2.05, −0.32]), had fewer respiratory symptoms (pooled RD −0.15 [95% CI −0.33, −0.04]), particularly pneumonia (pooled RD −0.13 [95% CI −0.26, −0.04]), and less frequently presented with non-cardiogenic shock or SIRS (pooled RD −0.35 [95% CI −0.64, −0.07]), particularly SIRS (pooled RD −0.28 [95% CI −0.46, −0.02]). Compared with patients admitted during the *Alpha* era, patients admitted during the *Omicron* era were also younger (pooled ES −1.6 years [95% CI −2.5, −0.8]) and less frequently presented with SIRS (pooled RD −0.18 [95% CI −0.30, −0.05]).

### Laboratory data

**Fig. 3:**
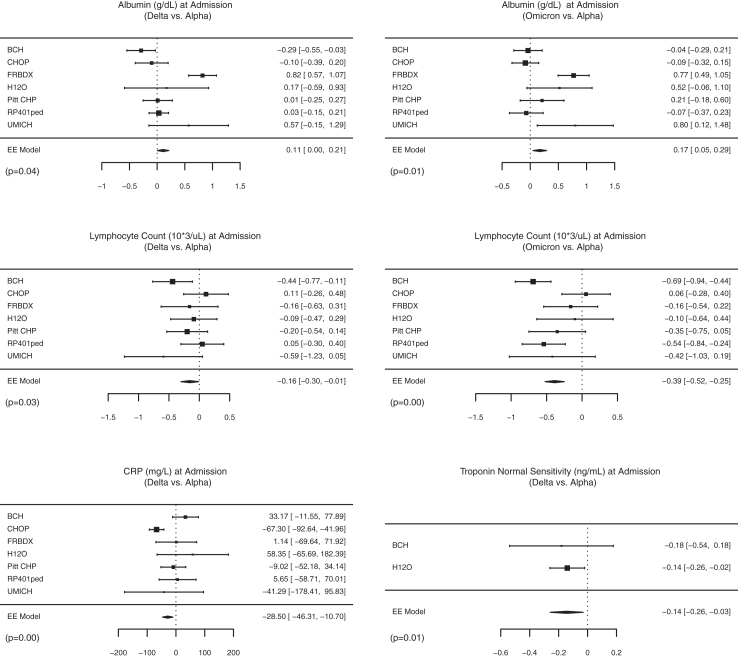
Significant differences in laboratory characteristics at admission according to SARS-CoV-2 variant eras by forest plots. Effect sizes and 95% confidence intervals are showed for each site using squared points and bars, respectively. Diamonds represent the pooled effect size and 95% confidence intervals. CRP: C-reactive protein; EE: exact effect.

**Fig. 4:**
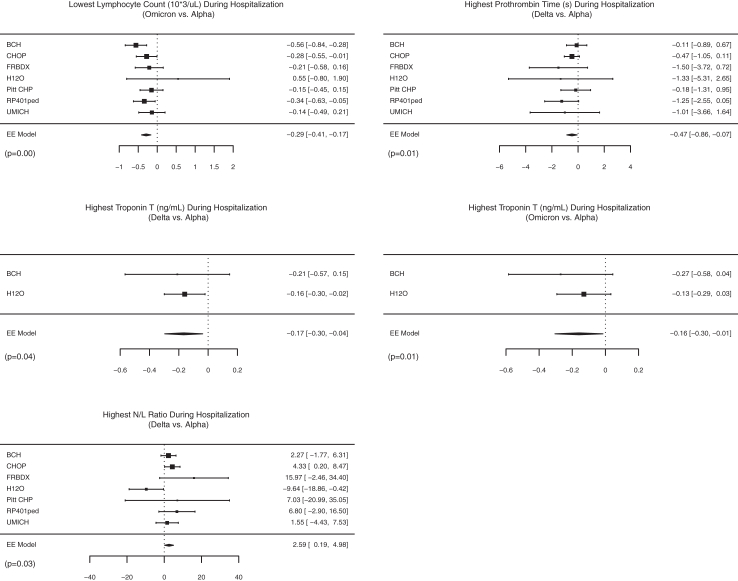
Significant differences in laboratory characteristics during hospitalization according to SARS-CoV-2 variant eras by forest plots. Effect sizes and 95% confidence intervals are showed for each site using squared points and bars, respectively. Diamonds represent the pooled effect size and 95% confidence intervals. EE: exact effect; N/L: neutrophile/lymphocyte ratio.

Laboratory characteristics for the whole cohort and across SARS-CoV-2 eras are shown in Fig. 3, Fig. 4, as well as Supplemental Tables S3 and S4. Overall, complete blood count abnormalities included abnormal white blood count (29%), lymphopenia (10%), anaemia (27%), and thrombocytopenia (22%). Coagulation abnormalities were seen in 25% and electrolyte abnormalities in 34%, including hyponatremia in 22%. Kidney dysfunction occurred in 21% and liver dysfunction in 11%.

Compared with patients admitted during the *Alpha* era, patients admitted during the *Delta* era had significantly lower absolute lymphocyte count (pooled ES –0.16 × 10^9^/uL [95% CI −0.30, −0.01]), C-reactive protein (CRP, pooled ES −28.5 mg/L [95% CI −46.3, −10.7]), and normal-sensitivity troponin-T (pooled ES −0.14 ng/mL [95% CI −0.26, −0.03]), as well as higher albumin at admission (pooled ES 0.1 g/dL [95% CI 0.01, −0.2]). The highest troponin-T and highest prothrombin time during hospitalization were also significantly lower in *Delta* patients (pooled ES −0.17 ng/mL [95% CI −0.30, −0.04] and pooled ES −0.5 s [95% CI −0.8, −0.1]). Compared with patients admitted during the *Alpha* era, those admitted during the *Omicron* era had lower lymphocyte counts (pooled ES −0.39 × 10^9^/uL [95% CI −0.52, −0.25]) and higher albumin at admission (pooled ES 0.2 g/dL [95% CI 0.1, 0.3]). During hospitalization, patients admitted during the *Omicron* era also had lower lymphocyte count (pooled ES –0.29 × 10^9^/uL [95% CI −0.41, −0.17]), higher neutrophil/lymphocyte ratio (pooled ES 2.5 [95% CI 0.2, 5.0]), and lower normal-sensitivity troponin-T (pooled ES −0.16 ng/mL [95% CI −0.30, −0.01]).

### Patient-level outcomes

Patient-level outcomes are shown in Table 2. Half of the cohort (51%) was admitted to the ICU. Diuretics were administered in 24% of patients, and anticoagulation therapy in 53%. Vasoactive/inotropic support was initiated in 12%, and 8% received supplemental oxygen or MV in 8%. Cardiac arrest occurred in three patients (1%), and two patients (1%) required ECMO. Coronary aneurysms were observed in 10% and coronary artery thrombosis in 1%. Forty-four percent had the composite adverse cardiovascular outcome. The pooled mean length of hospitalization was 7.3 days (pooled SD 6.8). No patients died during the study period.

**Table 2:** Patient-level outcomes in patients with MIS-C according to SARS-CoV-2 era.

Variable	Total, N = 436^a^	MIS-C during A*lpha* era, N = 275	MIS-C during *Delta* era, N = 87	MIS-C during *Omicron* era, N = 74	*Delta* compared to *Alpha*		*Omicron* compared to *Alpha*	
					Pooled RD or ES (95% CI)	P value	Pooled RD or ES (95% CI)	P value
ICU admission, N (%)	222 (50.9)	151 (54.9)	40 (45.98)	31 (41.89)	−0.028 (−0.119, 0.042)	0.456	0.026 (−0.186, 0.139)	1.000
Diuretic therapy, N (%)	107 (24.5)	70 (25.5)	17 (19.5)	20 (27.0)	−0.051 (−0.142, 0.023)	0.201	0.083 (0.048, 0.232)	0.454
Anticoagulation therapy, N (%)	233 (53.4)	175 (63.6)	38 (43.7)	20 (27.0)	0.013 (−0.208, 0.085)	1.000	−0.190 (−0.370, −0.037)	**0.008**
Sedation or muscle relaxant, N (%)	111 (25.5)	87 (31.6)	16 (18.4)	8 (10.1)	−0.021 (−0.110, 0.045)	0.524	−0.025 (−0.257, 0.053)	0.588
Vasoactive/inotropic support, N (%)	53 (12.2)	33 (12.0)	11 (12.6)	9 (12.2)	−0.008 (−0.058, 0.059	1.000	0.000 (−0.076, 0.105)	1.000
O2 supplementation or MV, N (%)	33 (7.6)	22 (8.0)	6 (6.9)	5 (6.8)	0.002 (−0.060, 0.065)	1.000	−0.048 (−0.149, 0.057)	0.632
Cardiac arrest, N (%)	3 (0.7)	3 (1.1)	0 (0.0)	0 (0.0)	−0.007 (−0.038, 0.041)	0.858	−0.007 (−0.046, 0.055)	0.964
ECMO, N (%)	2 (0.5)	2 (0.7)	0 (0.0)	0 (0.0)	0.000 (−0.034, 0.046)	1.000	−0.004 (−0.042, 0.058)	1.000
Coronary aneurysm, N (%)	42 (9.6)	27 (9.8)	6 (6.9)	9 (12.2)	−0.030 (−0.080, 0.057)	0.612	0.069 (−0.031, 0.193)	0.338
Coronary artery thrombosis or myocardial infarct, N (%)	3 (0.7)	3 (1.1)	0 (0.0)	0 (0.0)	−0.009 (−0.037, 0.042)	0.825	−0.009 (−0.046, 0.056)	0.864
Composite adverse cardiovascular outcome^b^, N (%)	193 (44.3)	130 (47.3)	33 (38.6)	30 (40.5)	−0.079 (−0.215, 0.062)	0.321	−0.013 (−0.257, 0.147)	1.000
Length of hospitalization, days, pooled mean (pooled SD) [N]	7.7 (6.6) [598]	8.1 (7.5) [383]	7.1 (4.4) [111]	7.1 (4.7) [104]	−1.345 (−2.287, −0.403)	**0.005**	−0.988 (−1.992, 0.016)	0.054
Mortality, N (%)	0 (0.0)	0 (0.0)	0 (0.0)	0 (0.0)	–	–	–	–

A detailed definition of the variables based on EHR data or ICD-10 codes is reported as Supplementary Material.

Aggregate counts and summary statistics for the total sample and the MIS-C era subgroups were calculated for descriptive purposes only. Meta-analyses were computed by pooling risk differences (RD, categorical variables) or effect sizes (ES, continuous variables) and their 95% confidence intervals (CIs) previously calculated at site-level.

ECMO: Extracorporeal Membrane Oxygenation; ES: effect size; ICU: intensive care unit; MIS-C: multisystem inflammatory syndrome; MV: mechanical ventilation; O2: oxygen; RD: risk difference; SD: standard deviation.

a ICD-10-code-based data from one centre, which did not pass the quality check, were excluded from analysis, thereby reducing sample size.

b Composite cardiovascular outcome: ventricular dysfunction, heart failure or cardiogenic shock (ICD-10 codes), inotropic/vasoactive drugs (EHR data), coronary aneurysm (ICD-10 codes), major arrhythmias (ICD-10 codes) cardiac arrest (EHR data and ICD-codes), VA-ECMO (EHR data).

Compared with patients admitted during the *Alpha* era, those admitted during the *Omicron* era less frequently received anticoagulation therapy (pooled RD −0.17 [95% CI −0.34, −0.03]). Length of hospitalization was shorter in patients admitted during the *Delta* versus *Alpha* eras (−1.3 days [95% CI −2.3, −0.4]). There were no other significant differences in patient-level outcome across eras.

### Sensitivity analyses

Sensitivity analyses confirmed the above-mentioned statistically significant differences, with the addition that prothrombin time at admission was lower in *Delta* versus *Alpha* (pooled ES −0.4 s [95% CI −0.8, −0.01]), and white blood count and D-dimer were lower in *Omicron* versus *Alpha* (pooled ES −1.79 × 10^9^/uL [95% CI −3.49, −0.09] pooled ES −884 ng/mL [95% CI −1747, −21]) (Supplemental Tables S5–S7).

## Discussion

With evolution of SARS-CoV-2 variants, therapeutic approaches, and population immunity, better understanding of the changing manifestations of COVID-19-associated diseases may improve clinical risk-stratification. This EHR-based study, which brings together data from a large cohort of patients with MIS-C from four countries across two continents, showed that MIS-C clinical and laboratory characteristics vary according to SARS-CoV-2 variant eras. We found that patients admitted during the *Delta* and *Omicron* eras were younger and less sick than those admitted in the *Alpha* era. Specifically, patients admitted during the *Alpha* era versus subsequent variant eras had more respiratory involvement, shock, and SIRS; higher CRP, absolute lymphocyte count, and troponin levels; lower albumin; and longer hospitalization.

Our retrospective multicentre study, using data extracted from the EHR from sites in the U.S. and Europe, adds to the evidence that COVID-19 phenotypes differ across SARS-CoV-2 eras.^4^^,^^18^, ^19^, ^20^ One recent study showed that paediatric patients with acute COVID-19 and laboratory-proven *Delta* or *Omicron* variants were more likely to have fever and respiratory symptoms than patients affected by other strains.^20^ A large multicentre study in children with acute COVID-19 showed that severe illness was significantly less common during the *Omicron* era versus the *Alpha* and *Delta* eras.^4^ This study also showed that MIS-C occurred more frequently during the *Alpha* era,^4^ consistent with other reports.^7^^,^^21^^,^^22^ In this context, recent studies have focused on investigating if MIS-C phenotypes may vary as well, based on SARS-CoV-2 variants.^6^, ^7^, ^8^^,^^10^, ^11^, ^12^, ^13^ Compared with EHR studies that selected patients with COVID-19-related conditions using diagnostic codes as the inclusion criteria, our study design had the advantage of patient selection using lists of patients with MIS-C validated by MIS-C experts at the site level.

Consistent with early reports,^8^ we showed that patients with MIS-C who were admitted during *Delta* and *Omicron* eras were younger than those admitted in the *Alpha* era. This may be related to differences in variant pathogenicity with predilection for younger patients for the recent variants, differences in the host immune response to the variant, and vaccination status.^23^^,^^24^ Particularly in the U.S. at the time of *Delta* and *Omicron* eras, older children may have received SARS-CoV-2 vaccination, which has been proven to protect against MIS-C.^25^^,^^26^ Data from Israel first showed that receiving even one dose of vaccine was associated with a lower risk of MIS-C.^25^ A large multicentre case-control study subsequently showed that patients with MIS-C were less likely to have been fully vaccinated compared with hospitalised controls.^26^ Another recent study showed that all but one MIS-C patient during the *Delta* period were found to be unvaccinated, with an estimated vaccine effectiveness of 94% (95% CI 55–99%). Interestingly, data from South Africa, which included all unvaccinated individuals, showed no difference in age among MIS-C groups based on variant.^9^ Further studies will likely be needed to clarify this aspect.

Analysis of clinical characteristics showed that patients admitted during the *Delta* era less frequently had respiratory involvement, in particular pneumonia. Whereas smaller studies showed inconsistent data on respiratory symptoms,^9^, ^10^, ^11^, ^12^ our findings are similar to those reported by a large CDC-based study^6^ and a large recent North American report.^8^ In our study, patients admitted during the *Delta* or *Omicron* eras were also less likely to present with non-cardiogenic shock or SIRS; interestingly, the prevalence of cardiogenic shock did not change across eras. This represents novel results compared to previous analyses, which pooled all types of shock together.^6^^,^^8^^,^^10^ Changes in cardiorespiratory symptoms over time may be due to different host immune responses to different strains, with milder inflammation response to more recent variants,^23^^,^^24^ potentially modulated by the vaccination status,^23^^,^^24^ or may be a consequence of earlier and more effective treatment approaches in the more recent era, such as an increased use of steroids.^8^

In terms of illness severity and outcomes, previous studies showed less frequent ICU admission and shorter hospital length of stay among patients admitted in the post-*Alpha* eras.^6^^,^^7^ Our study was consistent in showing a shorter length of hospitalization for patients admitted in the post-*Alpha* eras, but did not find any significant difference in the rates of ICU admission. This may be due to differences in institutional practices on the criteria for ICU admission, or to changes in clinical practice following the first era. Cardiovascular outcomes, when analysed as dichotomous measures of the presence or absence of ventricular dysfunction, major arrhythmias, coronary aneurysm, cardiac arrest, ECMO, or as a composite measure, did not significantly differ across eras. However, we showed that patients admitted during the *Delta* or *Omicron* eras had significantly lower troponin levels compared with those admitted during the *Alpha* era. This finding is consistent with results of a smaller study from Israel^7^ and a larger CDC data-based study,^6^ suggesting that cardiovascular outcomes may be milder in post-*Alpha* eras.

The reason why patients admitted during *Alpha* were more frequently treated with anticoagulants is unclear. It may be related to less severe cardiovascular outcomes in post-*Alpha* eras, and to lower D-dimer levels as suggested by the sensitivity analyses. In addition, changes in thromboprophylaxis over time may have been affected by refinement of criteria for the use of anticoagulants versus acetylsalicylic acid (aspirin) in national and international guidelines.^27^^,^^28^ The possibility of reduced practice variation due to publication of guidelines is supported by early reports showing wide variability in use of anticoagulation or antiplatelet therapy, with some of the centres administering anticoagulation to most of the patients.^1^^,^^16^^,^^29^^,^^30^

Our study also showed how EHR data may be leveraged effectively to answer clinical questions without the need for sharing protected health information and while considering centre-level variability. Laboratory test results and the other EHR-based variables have the advantage of being directly extracted without any human involvement, limiting any typo-errors, and enabling all results to be considered for our analysis. This collaborative multicentre infrastructure may be used to identify trends in epidemiology, disease phenotype, and treatment of diverse diseases requiring hospitalization. Once algorithms have been created and validated across sites, they may be deployed rapidly and serve as an effective easily-available surveillance tool to inform clinical care and public health policy.

Our study has limitations. Its retrospective design carries risks of missing data and reporting bias. Although the EHR-based automatic data extraction might have helped in decreasing the amount of missing data, we could not control for data not entered in the EHR and for reporting bias such as more severe symptoms possibly being entered more frequently than those less severe. Additionally, our study used the 4CE infrastructure, developed for COVID-19 research, which did not include specific data on MIS-C including treatments such as intravenous immunoglobulin or corticosteroids. Moreover, it was not possible to reliably extract vaccination status from the EHR, since ICD-10 codes were available only for the last part of the study period. Coding practices likely vary at each site; however, to avoid missing codes (e.g., European-specific ICD-10 codes), we performed an extensive quality check by manually reviewing all ICD-10 codes attributed to patients with MIS-C at each site, and–to partially control for a site-effect–we first calculated the risk difference within institution and only as a second step we computed a meta-analysis across sites. Microbiology data on variants were not available; thus, SARS-CoV-2 eras were defined based on epidemiological assumptions; however, this approach has been widely used by previous studies,^6^, ^7^, ^8^, ^9^, ^10^, ^11^, ^12^ and sensitivity analyses were performed to improve the reliability of our inferences. Moreover, although we included international data across two continents, representativeness of and within countries is limited and generalizability to other geographic areas will need to be confirmed. An additional limitation was the use of different criteria for MIS-C based on the nation of the institution. Finally, the meta-analysis method we employed to account for small sample sizes might have been overly conservative, as stated by the authors^17^; however, we preferred a conservative approach to avoid overstatements. Given the small size of the subgroups, further stratification analyses—e.g., based on age-subgroups—were not possible.

Despite these limitations, we believe our study using real-world data adds valuable information in characterizing different MIS-C phenotypes across SARS-CoV-2 eras and continents. MIS-C clinical and laboratory characteristics vary according to SARS-CoV-2 variant eras, with patients in the *Delta* and *Omicron* eras being younger and having a less severe presentation than those in the *Alpha* era as indicated by clinic manifestations, laboratory data, and patient-level-outcome. EHR-based aggregate data may be effectively leveraged to investigate rare complications of diseases in the setting of the pandemic and used to identify patterns of disease variation or severity over time.

## Contributors

FS, AGS, SM, TC, FTB, GSO, KM, JWN, and PA contributed to the design and conceptualization of the study. FS, AGS, SM, DAH, CLB, NGB, TGG, RI, KFK, SEM, KDM, TMA, CMR, MM, KM, MPJ, MAS, AS, DMT, GV, SV, ZX, and PA contributed to data collection and verified the underlying data reported in the manuscript. FS, AGS, SM, XL, VNR, TC, FTB, GSO, CS, EB, AD, ME, KFK, JLG, SEM, TMA, MM, MAS, AMS, DMT, XW, JMZ, and PA contributed to data analysis or interpretation. SEM supplied grant funding for the work. All authors contributed to drafting the work or revising it critically for important intellectual content and approved the final version. All authors had full access to all the data, accept full responsibility of ensuring accuracy or integrity of any part of the work, approved the final version of the manuscript and agreed to submit it for publication.

## Data sharing statement

Summary data according to site and SARS-CoV-2 era are publicly available through the study visualization tool (https://avillachlab.shinyapps.io/a1l1non3/). The analytic code is publicly available on GitHub (https://github.com/covidclinical/PhaseX.2SqlDataExtraction/tree/main/Extras, https://github.com/covidclinical/Phase2.2MISCRPackage).

## Declaration of interests

The authors have no conflicts of interests to declare related to the content of this manuscript. JNW has research grant funding from National Heart, Lung, and Blood Institute (NHLBI), the Department of Defense, the Centres for Disease Control (CDC), and Pfizer; has been a consultant for Pfizer; chaired the Independent Events Adjudication Committees for Novartis, Pfizer, and Bristol-Myer-Squibb; and received honoraria from Daiichi Sankyo for service on the Steering Committee of the ENNOBLE-ATE Trial and from UpToDate. GSO has research grant funding from the National Institute of Environmental Health Sciences (NIEHS), National Institutes of Health (NIH), and from the National Cancer Institute (NCI). DAH has research grant funding from the National Center for Advancing Translational Sciences (NCATS). TGG has research grant funding from the Institute of Health Carlos III, the European Regional Development Fund (ERDF), the National Institute of Environmental Health Sciences (NIEHS), National Institutes of Health (NIH), and National Cancer Institute (NCI). KFK has research grant funding from the National Institute of Child Health and Human Development (NIHCD). SEM has research grant funding from the National Center for Advancing Translational Sciences (NCATS). AD has research grant funding from Pfizer. DMT has research grant funding from NIH. AMS has research grant funding from the National Heart, Lung, and Blood Institute (NHLBI) and from the National Center for Advancing Translational Sciences (NCATS). GV has internal research funding from the Centre Hospitalier Universitaire de Bordeaux. ZX has research grant funding from the National Institute of Neurological Disorders and Stroke (NINDS). None of these funding sources had any role in supporting the study design; in the collection, analysis, and interpretation of data; in the writing of the report; or in the decision to submit the paper for publication. All the other authors have no conflicts of interests to declare.

## Appendix A Supplementary data

Supplementary Materials

Members List
