# Supplementary material for: Clinical phenotypes and outcomes in children with multisystem inflammatory syndrome across SARS-CoV-2 variant eras: a multinational study from the 4CE consortium
Source: eClinicalMedicine. 2023 Sep 14;64:102212. doi: 10.1016/j.eclinm.2023.102212 (PMC10511777; doi:10.1016/j.eclinm.2023.102212)
Supplement: Supplementary Materials [file mmc1.pdf]

# Appendix

# Clinical phenotypes and outcomes in children with multisystem inflammatory syndrome across SARS-CoV-2 variant eras: a multinational study from the 4CE Consortium

Francesca Sperotto MD, PhD, Alba Gutiérrez-Sacristán PhD, Simran Makwana MS, Xiudi Li PhD, Valerie N Rofeberg ScM, Tianxi Cai ScD, Florence T Bourgeois MD, MPH, Gilbert S Omenn MD, PhD, David A Hanauer MD, MS, Carlos Sáez PhD, Clara-Lea Bonzel MSc, Emily Bucholz MD, PhD, MPH, Audrey Dionne MD, Matthew D Elias MD, Noelia García-Barrio MS, Tomás González González MD, Richard W Issitt DClinP, Kernan F Kate MD, Jessica Laird-Gion MD, Sarah E Maidlow AA, Kenneth D Mandl MD, MPH, Taha Mohseni Ahooyi PhD, Cinta Moraleda MD,PhD, Michele Morris BA, Karyn L Moshal MBChB, FRCPCH, Miguel Pedrera-Jiménez MS, Mohsin A Shah MSc, Andrew M South MD, MS, Anastasia Spiridou PhD, Deanne M Taylor PhD, Guillaume Verdy MSc, Shyam Visweswaran MD, PhD, Xuan Wang PhD, Zongqi Xia MD, PhD, Joany M Zachariasse MD, PhD, The Consortium for Clinical Characterization of COVID-19 by EHR (4CE), Jane W Newburger MD, MPH, Paul Avillach MD, PhD

**Table of content**


- **Supplemental Table 1.** Contributing hospital details, including MIS-C definition criteria, number of contributing MIS-C patients to the study, and obfuscation thresholds.
- **Supplemental Table 2.** Case definitions for SARS-CoV-2-associated Multisystem Inflammatory Syndrome in Children.
- **Supplemental Table 3.** Additional ICD-10 code-based characteristics of patients with MIS-C according to SARS-CoV-2 era.
- **Supplemental Table 4.** Laboratory values at admission and worst during hospitalization in patients with MIS-C according to SARS-CoV-2 era.
- **Supplemental Table 5.** Sensitivity analyses assessing demographic and clinical characteristics of patients with MIS-C according to SARS-CoV-2 variant era (with cut off dates shifted by 2 weeks later).
- **Supplemental Table 6.** Sensitivity analyses assessing patient-level outcomes in patients with MIS-C according to SARS-CoV-2 era (with cut off dates shifted by 2 weeks later).
- **Supplemental Table 7.** Sensitivity analyses assessing laboratory values at admission and worst during hospitalization in patients with MIS-C according to SARS-CoV-2 era (with cut off dates shifted by 2 weeks later).
- **Supplemental Table 8.** Laboratory test details, including units, Logical Observation Identifier Names and Codes (LOINC) code, and worst value.
- **Supplemental Figure 1**. Schematics of the 4CE Consortium federated approach to develop and conduct analyses across sites.
- **Supplemental Methods 1**. Data quality check procedure.
- **Supplemental Methods 2.** List of ICD-10 codes of interest.
- **Supplemental Methods 3.** List of EHR 4CE data definitions.
- **Supplemental Information 1.** The Consortium for Clinical Characterization of COVID-9 by EHR (4CE) Members

**Supplemental Table 1.** Contributing hospital details, including MIS-C definition criteria, number of contributing MIS-C patients to the study, and obfuscation thresholds.

| **Country** | **Center** | **Center official abbreviation** | **MIS-C definition criteria*** | **Obfuscation threshold**** | **N of MIS-C patients** |
|---|---|---|---|---|---|
| United States of America | University of Michigan, Ann Arbor, Michigan | UMICH | CDC | none | 31 |
| | Boston Children's Hospital, Boston, Massachusetts | BCH | CDC | none | 137 |
| | The Children's Hospital of Philadelphia, Philadelphia, Pennsylvania | CHOP | CDC | none | 162 |
| | University of Pittsburgh Medical Center, Pittsburgh, Pennsylvania | PittCHP | CDC | none | 54 |
| United Kingdom | Great Ormond Street Hospital for Children, London | RP401ped | RCPCH | N ≤3 | 143 |
| Spain | Hospital Universitario 12 de Octubre, Madrid | H12O | WHO | none | 25 |
| France | Bordeaux University Hospital, Bordeaux | FRBDX | WHO | N ≤1 | 46 |

* MIS-C definition criteria are reported in **Supplemental Table 2.**
** The obfuscation threshold refers to the minimum patient count that will be reported by a site to ensure patients' privacy.
CDC: Centers for Disease Control and Prevention; MIS-C: multisystem inflammatory syndrome in children; N: number; RCPCH: Royal College of Paediatrics and Child Health; WHO: World Health Organization.

**Supplemental Table 2.** Case definitions for SARS-CoV-2-associated Multisystem Inflammatory Syndrome in Children.

| Royal College of Paediatrics and Child Health, United Kingdom | Centers for Disease Control and Prevention (CDC), United States | World Health Organization (WHO) |
|---|---|---|
| A child presenting with persistent fever (>38.5ºC), inflammation (neutrophilia, elevated CRP, and lymphopenia) and evidence of single or multi-organ dysfunction (shock, cardiac, respiratory, kidney, gastrointestinal, or neurological disorder) with additional features*.<br><br>This may include children fulfilling full or partial criteria for KD.<br><br>Exclusion of any other microbial cause, including bacterial sepsis, staphylococcal or streptococcal shock syndromes, infections associated with myocarditis such as enterovirus (waiting for results of these investigations should not delay seeking expert advice).<br><br>SARS-CoV-2 RT-PCR test results may be positive or negative.<br><br>*<u>Additional features:</u><br>Clinical:<br>Most: oxygen requirement, hypotension<br>Some: abdominal pain, confusion, conjunctivitis, cough, diarrhoea, headache, lymphadenopathy, mucus membrane changes, neck swelling, rash, respiratory symptoms, sore throat, swollen hands and feet, syncope vomiting;<br>Laboratory:<br>All: abnormal fibrinogen, high D-dimers, high ferritin, hypoalbuminemia;<br>Some: acute kidney injury, anaemia, thrombocytopenia, coagulopathy, high IL-10, high IL-6, proteinuria, high CK, high LDH, high TG, high troponin, transaminitis;<br>Imaging:<br>Echo and ECG: myocarditis, valvulitis, pericardial effusion, coronary artery dilation;<br>CXR: patchy symmetrical infiltrates, pleural effusion;<br>Abdo USS: colitis, ileitis, lymphadenopathy, ascites, hepatosplenomegaly;<br>CT chest: as for CXR. May demonstrate coronary artery abnormalities if with contrast. | An individual aged <21 years presenting with fever*, laboratory evidence of inflammation**, and evidence of clinically severe illness requiring hospitalization, with multisystem (≥2) organ involvement (cardiac, renal, respiratory, hematologic, gastrointestinal, dermatologic, or neurological);<br><br>**AND**<br><br>No alternative plausible diagnoses;<br><br>**AND**<br><br>Positive for current or recent SARS-CoV-2 infection by RT-PCR, serology, or antigen test, or COVID-19 exposure within 4 weeks prior to the onset of symptoms.<br><br>*Fever ≤38°C for ≥24 hours, or report of subjective fever lasting ≥24 hours.<br>**Including, but not limited to, one or more of the following: an elevated CRP, ESR, fibrinogen, procalcitonin, d-dimer, ferritin, LDH, or IL-6, elevated neutrophils, reduced lymphocytes, and low albumin.<br><br><u>Additional comments:</u><br>Some individuals may fulfil or partial criteria for KD but should reported if they meet the case definition for MIS-C;<br>Consider MIS-C in any pediatric death with evidence of SARS-Cov-2 infection. | Children and adolescents 0–19 years of age with fever ≥ 3 days;<br><br>**AND** <u>two</u> of the following:<br><br>1. Rash or bilateral non-purulent conjunctivitis or [illegible] cutaneous inflammation signs (oral, hands or fe[illegible]<br>2. Hypotension or shock.<br>3. Features of myocardial dysfunction, pericarditis, valvulitis, or coronary abnormalities (including echo findings or elevated Troponin/NT-proBNP),<br>4. Evidence of coagulopathy (by PT, PTT, elevated d-Dimers).<br>5. Acute gastrointestinal problems (diarrhoea, vomiting, or abdominal pain).<br><br>**AND**<br><br>Elevated markers of inflammation such as ESR, C-reactive protein, or procalcitonin.<br><br>**AND**<br><br>No other obvious microbial cause of inflammation, including bacterial sepsis, staphylococcal or streptococcal shock syndromes.<br><br>**AND**<br><br>Evidence of COVID-19 **(**RT-PCR, antigen test or serology positive), or likely contact with patients with COVID-19.<br><br>Consider this syndrome in children with features of typical or atypical KD or toxic shock syndrome |

APTT: activated partial thromboplastin time; CK: creatine kinase; COVID-19: coronavirus disease 2019; CXR: chest X-ray; CRP, C-reactive protein; echo: echocardiography; ESR: erythrocyte sedimentation rate; IL: interleukin; KD: Kawasaki disease; LDH: lactic acid dehydrogenase; MIS-C: multisystem inflammatory syndrome in children; NT-proBNP, N-terminal pro–B-type natriuretic peptide; PT: prothrombin time; PTT: partial thromboplastin time; RT-PCR: reverse transcriptase–polymerase chain reaction; SARS-CoV-2: severe acute respiratory syndrome coronavirus 2; TG: triglycerides

**Supplemental Table 3.** Additional ICD-10-codes based characteristics of patients with MIS-C according to SARS-CoV-2 eras.

| Variable | Total N=436* | MIS-C during *Alpha* era N=275 | MIS-C during *Delta* era N=87 | MIS-C during *Omicron* era N=74 | ***Delta*** **compared to *Alpha*** | | ***Omicron*** **compared to *Alpha*** | |
|---|---|---|---|---|---|---|---|---|
| | | | | | **Pooled RD (95% CI)** | **P value** | **Pooled RD (95% CI)** | **P value** |
| Dehydration, N (%) | 104 (23·8) | 52 (18·9) | 28 (32·2) | 24 (32·4) | 0·055 (-0·024, 0·183) | 0·205 | 0·066 (-0·062, 0·205) | 0·658 |
| Fluid overload/generalized oedema, N (%) | 54 (12·4) | 34 (12·4) | 12 (13·8) | 8 (10·8) | 0·011 (-0·076, 0·108) | 1·000 | 0·000 (-0·071, 0·115) | 1·000 |
| Ascites, N (%) | 56 (12·8) | 36 (13·1) | 12 (13·8) | 7 (9·5) | 0·012 (-0·049, 0·172) | 1·000 | 0·057 (-0·110, 0·159) | 0·805 |
| Hepatosplenomegaly, N (%) | 41 (9·4) | 25 (9·1) | 8 (9·2) | 8 (10·8) | 0·031 (-0·060, 0·120) | 0·722 | 0·048 (-0·061, 0·146) | 0·980 |
| Acidosis, N (%) | 41 (9·4) | 25 (9·1) | 5 (5·7) | 11 (14·9) | -0·014 (-0·081, 0·084) | 1·000 | 0·124 (-0·006, 0·270) | 0·075 |
| Alkalosis, N (%) | 14 (3·2) | 11 (4·0) | 1 (1·1) | 2 (2·7) | -0·018 (-0·055, 0·046) | 0·719 | 0·000 (-0·038, 0·066) | 1·000 |
| Abnormal WBC, N (%) | 125 (28·7) | 89 (32·4) | 18 (20·7) | 18 (24·3) | -0·043 (-0·200, 0·068) | 0·547 | -0·100 (-0·255, 0·073) | 0·484 |
| Elevated WBC, N (%) | 25 (5·7) | 18 (6·5) | 3 (3·4) | 4 (5·4) | 0·000 (-0·044, 0·056) | 1·000 | -0·001 (-0·074, 0·073) | 1·000 |
| Decreased WBC, N (%) | 13 (3·0) | 9 (3·3) | 2 (2·3) | 2 (2·7) | 0·010 (-0·028, 0·067) | 0·716 | 0·007 (-0·051, 0·081) | 0·917 |
| Lymphocytopenia, N (%) | 44 (10·1) | 28 (10·2) | 7 (8·0) | 9 (12·2) | 0·000 (-0·063, 0·067) | 1·000 | 0·036 (-0·081, 0·163) | 0·926 |
| Other abnormal WBC, N (%) | 52 (11·9) | 39 (14·2) | 8 (9·2) | 5 (6·8) | -0·015 (-0·102, 0·058) | 0·768 | -0·040 (-0·218, 0·021) | 0·190 |
| Anaemia, N (%) | 117 (26·8) | 67 (24·7) | 20 (23·0) | 30 (40·5) | -0·084( -0·207, 0·168) | 0·910 | 0·180 (-0·216, 0·396) | 0·917 |
| Thrombocytopenia, N (%) | 95 (21·8) | 58 (21·1) | 14 (16·1) | 23 (31·1) | -0·076 (-0·157, 0·075) | 0·540 | 0·165 (-0·080, 0·456) | 0·959 |
| Coagulation abnormalities, N (%) | 110 (25·2) | 85 (31·0) | 14 (16·1) | 11 (14·9) | -0·085 (-0·165, 0·035) | 0·152 | -0·110 (-0·245, 0·004) | 0·065 |
| Electrolytes abnormalities, N (%) | 147 (33·7) | 93 (33·8) | 27 (31·0) | 27 (36·5) | -0·063 (-0·190, 0·068) | 0·384 | 0·131 (-0·327, 0·300) | 0·810 |
| Hypokalaemia, N (%) | 60 (13·8) | 42 (15·3) | 9 (10·3) | 9 (12·2) | -0·089 (-0·157, 0·073) | 0·432 | -0·058 (-0·136, 0·044) | 0·268 |
| Hyponatremia, N (%) | 97 (22·2) | 56 (20·4) | 21 (24·1) | 20 (27·0) | -0·065 (-0·173, 0·066) | 0·325 | 0·109 (-0·294, 0·284) | 0·968 |
| Elevated LDH, N (%) | 32 (7·3) | 25 (9·1) | 3 (3·4) | 4 (5·4) | -0·060 (-0·122, 0·009) | 0·099 | -0·078 (-0·173, 0·027) | 0·170 |
| Hyperglycaemia, N (%) | 21 (4·8) | 15 (5·4) | 1 (1·1) | 5 (6·8) | -0·035 (-0·093, 0·030) | 0·931 | 0·023 (-0·075, 0·134) | 1·000 |

*ICD-10 code-based data from one center, which did not pass a quality check, were excluded from analysis, thereby reducing sample size.

Aggregate counts and summary statistics for the total sample and the MIS-C era subgroups were calculated for descriptive purposes only. Meta-analyses were computed by pooling risk differences (RD, categorical variables) or effect sizes (ES, continuous variables) and their 95% confidence intervals (CIs) previously calculated at site-level. A detailed definition of the variables based on EHR data or ICD-10 codes is reported as Supplementary Material.

LDH: lactate dehydrogenases; MIS-C: multisystem inflammatory syndrome; RD: risk difference; SD: standard deviation; WBC: white blood count.

**Supplemental Table 4.** Laboratory values at admission and worst during hospitalization in patients with MIS-C according to SARS-CoV-2 eras.

| Variable | Total N=598 | MIS-C during *Alpha* era N=383 | MIS-C during *Delta* era N=111 | MIS-C during *Omicron* era N=104 | ***Delta* compared to *alpha*** | | ***Omicron* compared to *Alpha*** | |
|---|---|---|---|---|---|---|---|---|
| | | | | | **Pooled ES (95% CI)** | **P value** | **Pooled ES (95% CI)** | **P value** |
| **At admission** | | | | | | | | |
| WBC, x10⁹/uL, pooled mean (pooled SD) | 11·992 (7·029) [N=427] | 12·323 (7·211) [N=270] | 11·702 (7·436) [N=86] | 11·097 (5·689) [N=71] | -0·342 (-1·996,1·313) | 0·686 | -1·193 (-2·723,0·337) | 0·126 |
| Lymphocyte count, x10⁹/uL, pooled mean (pooled SD) | 1·155 (0·788) [N=525] | 1·224 (0·846) [N=332] | 1·08 (0·69) [N=105] | 0·984 (0·626) [N=88] | -0·157 (-0·302,-0·012) | **0·034** | -0·386 (-0·521,-0·25) | **<0·001** |
| Neutrophil count, x10⁹/uL, pooled mean (pooled SD) | 9·325 (5·78) [N=525] | 9·342 (5·743) [N=332] | 9·663 (6·493) [N=105] | 8·852 (4·999) [N=88] | 0·338 (-0·659,1·336) | 0·506 | -0·610 (-1·62,0·399) | 0·236 |
| N/L ratio, pooled mean (pooled SD) | 12·6 (15·4) [N=525] | 11·7 (11·5) [N=332] | 12·2 (10·3) [N=105] | 16·2 (28·1) [N=88] | 0·671 (-1·054,2·396) | 0·446 | 2·163 (-0·278,4·604) | 0·082 |
| Platelets count, x10⁹/uL, pooled mean (pooled SD) | 188·767 (90·119) [N=559] | 185·734 (87·887) [N=360] | 189·978 (85·704) [N=107] | 199·245 (103·068) [N=92] | 4·401 (-12·57,21·371) | 0·611 | 3·858 (-15·325,23·041) | 0·693 |
| C-reactive protein, mg/L, pooled mean (pooled SD) | 162·7 (102·2) [N=554] | 166·9 (102·3) [N=355] | 159·6 (106·5) [N=108] | 149·8 (96·5) [N=91] | -28·504 (-46·312,-10·695) | **0·002** | -6·037 (-26·739,14·664) | 0·568 |
| Albumin, g/dL, pooled mean (pooled SD) | 3·2 (0·6) [N=524] | 3·2 (0·6) [N=339] | 3·1 (0·6) [N=99] | 3·3 (0·6) [N=86] | 0·108 (0·004,0·212) | **0·042** | 0·171 (0·051,0·29) | **0·005** |
| D-dimer, ng/mL, pooled mean (pooled SD) | 4332·1 (7228·0) [N=443] | 4161·2 (6779·4) [N=296] | 4334·4 (7624·2) [N=83] | 5119·4 (8654·5) [N=64] | -402·698 (-1219·345,413·949) | 0·334 | -694·906 (-1520·45,130·639) | 0·099 |
| Prothrombin time, s, pooled mean (pooled SD) | 14·1 (2·7) [N=430] | 14·2 (2·9) [N=279] | 13·7 (2·4) [N=80] | 14·4 (2·3) [N=71] | -0·225 (-0·66,0·209) | 0·310 | -0·145 (-0·627,0·336) | 0·554 |
| Fibrinogen, mg/dL, pooled mean (pooled SD) | 556·1 (192·1) [N=397] | 546·9 (188·5) [N=258] | 559·3 (196·9) [N=76] | 590·1 (200·3) [N=63] | -9·989 (-43·518,23·541) | 0·559 | 16·077 (-21·004,53·158) | 0·395 |
| Ferritin, ug/L, pooled mean (pooled SD)11·988 (7·088) [N=402] | 1026·01 (1873·6) [N=465] | 1021·2 (2155·8) [N=300] | 923·7 (959·7) [N=87] | 1158·6 (1431·5) [N=78] | 41·046 (-149·647,231·739) | 0·673 | 166·386 (-15·97,348·742) | 0·074 |
| ALT, U/L, pooled mean (pooled SD) | 53·5 (173·4) [N=546] | 54·9 (210·6) [N=352] | 42·4 (32·6) [N=104] | 60·8 (88·8) [N=90] | -5·302 (-12·572,1·968) | 0·153 | 3·427 (-6·028,12·881) | 0·477 |
| AST, U/L, pooled mean (pooled SD) | 87·0 (405·6) [N=412] | 97·1 (514·1) [N=247] | 59·2 (39·6) [N=82·5] | 82·8 (172·0) [N=81·5] | -8·743 (-20·459,2·974) | 0·144 | -5·769 (-17·428,5·891) | 0·332 |
| Creatinine, mg/dL, pooled mean (pooled SD) | 0·67 (0·53) [N=559] | 0·67 (0·53) [N=358] | 0·62 (0·38) [N=109] | 0·70 (0·64) [N=92] | -0·063 (-0·145,0·019) | 0·131 | -0·078 (-0·174,0·018) | 0·110 |
| Troponin T, normal sensitivity, ng/mL, pooled mean (pooled SD)* | 0·19 (1·23) [N=144] | 0·27 (1·49) [N=95] | 0·08 (0·31) [N=19] | 0·04 (0·10) [N=30] | -0·144 (-0·258, -0·030) | **0·013** | -0·121 (-0·259, 0·018) | 0·088 |
| Troponin T, high sensitivity, ng/mL, pooled mean (pooled SD)* | 0·51 (1·30) [N=221] | 0·45 (0·98) [N=141] | 0·62 (1·89) [N=50] | 0·58 (1·35) [N=30] | -0·176 (-0·614,0·261) | 0·429 | -0·047 (-0·578,0·483) | 0·861 |
| **During hospitalization** | | | | | | | | |
| WBC, x10⁹/uL, highest, pooled mean (pooled SD) | 11·992 (7·029) [N=427] | 12·323 (7·211) [N=270] | 11·702 (7·436) [N=86] | 11·097 (5·689) [N=71] | -0·961 (-2·73,0·809) | 0·287 | -0·588 (-2·927,1·750) | 0·622 |
| Lymphocyte count, lowest, x10⁹/uL, pooled mean (pooled SD) | 1·155 (0·788) [N=524] | 1·224 (0·846) [N=331] | 1·08 (0·69) [N=105] | 0·984 (0·626) [N=88] | -0·066 (-0·186,0·054) | 0·282 | -0·291 (-0·414,-0·167) | **<0·001** |
| Neutrophil count, highest, x10⁹/uL, pooled mean (pooled SD) | 9·325 (5·78) [N=525] | 9·342 (5·743) [N=332] | 9·663 (6·493) [N=105] | 8·852 (4·999) [N=88] | 0·491 (-0·723,1·704) | 0·428 | 0·449 (-0·858,1·757) | 0·501 |

| | | | | | | | | |
|---|---|---|---|---|---|---|---|---|
| N/L ratio, highest, pooled mean (pooled SD) | 12·6 (15·4) [N=524] | 11·7 (11·5) [N=331] | 12·2 (10·3) [N=105] | 16·2 (28·1) [N=88] | 0·597 (-1·203,2·398) | 0·516 | 2·585 (0·189,4·981) | **0·034** |
| Platelets count, highest, x10$^9$/uL, pooled mean (pooled SD) | 188·767 (90·119) [N=559] | 185·734 (87·887) [N=360] | 189·978 (85·704) [N=107] | 199·245 (103·068) [N=92] | -9·227 (-39·391,20·937) | 0·549 | -3·678 (-38·183,30·827) | 0·835 |
| C-reactive protein, highest, mg/L, pooled mean (pooled SD) | 162·7 (102·2) [N=554] | 166·9 (102·3) [N=355] | 159·6 (106·5) [N=108] | 149·8 (96·5) [N=91] | -6·960 (-24·770,10·850) | 0·444 | -12·376 (-28·596,3·845) | 0·135 |
| Albumin, lowest, g/dL, pooled mean (pooled SD) | 3·2 (0·6) [N=524] | 3·2 (0·6) [N=339] | 3·1 (0·6) [N=99] | 3·3 (0·6) [N=86] | -0·003 (-0·090,0·084) | 0·941 | -0·042 (-0·136,0·053) | 0·387 |
| D-dimer, highest, ng/mL, pooled mean (pooled SD) | 4332·1 (7228·0) [N=443] | 4161·2 (6779·4) [N=296] | 4334·4 (7624·2) [N=83] | 5119·4 (8654·5) [N=64] | -453·516 (-1284·856,377·824) | 0·285 | -572·885 (-1316·09,170·327) | 0·131 |
| Prothrombin time, highest, s, pooled mean (pooled SD) | 14·1 (2·7) [N=430] | 14·2 (2·9) [N=279] | 13·7 (2·4) [N=80] | 14·4 (2·3) [N=71] | -0·467 (-0·862,-0·073) | **0·020** | -0·317 (-0·881,0·247) | 0·271 |
| Fibrinogen, highest, mg/dL, pooled mean (pooled SD) | 556·1 (192·1) [N=397] | 546·9 (188·4) [N=258] | 559·3 (196·9) [N=76] | 590·1 (200·3) [N=63] | 1·609 (-34·151, 37·369) | 0·930 | 12·337 (-23·032, 47·705) | 0·494 |
| Ferritin, highest, ug/L, pooled mean (pooled SD) | 1026·0 (1873·6) [N=465] | 1021·2 (2155·8) [N=300] | 923·7 (959·7) [N=87] | 1158·6 (1431·4) [N=78] | 55·907 (-177·187,289·001) | 0·638 | 143·211 (-106·78,393·201) | 0·262 |
| ALT, highest, U/L, pooled mean (pooled SD) | 53·5 (173·4) [N=546] | 54·9 (210·5) [N=352] | 42·4 (32·6) [N=104] | 60·8 (88·8) [N=90] | -11·405 (-23·91,1·101) | 0·074 | 3·893 (-10·122,17·908) | 0·586 |
| AST, highest, U/L, pooled mean (pooled SD) | 86·7 (405·6) [N=412] | 97·1 (514·1) [N=247] | 59·2 (39·5) [N=82·5] | 82·8 (172·0) [N=81·5] | -7·454 (-20·513,5·605) | 0·263 | -6·336 (-18·625,5·952) | 0·312 |
| Creatinine, highest, mg/dL, pooled mean (pooled SD) | 0·67 (0·53) [N=559] | 0·67 (0·53) [N=358] | 0·63 (0·38) [N=109] | 0·70 (0·64) [N=92] | -0·061 (-0·129,0·007) | 0·078 | -0·009 (-0·08,0·062) | 0·801 |
| Troponin T, normal sensitivity, highest, ng/mL, pooled mean (pooled SD)* | 0·19 (1·23) [N=144] | 0·27 (1·50) [N=95] | 0·08 (0·31) [N=19] | 0·04 (0·10) [N=30] | -0·167 (-0·296, -0·037) | **0·012** | -0·160 (-0·304, -0·015) | **0·031** |
| Troponin T, high sensitivity, highest, ng/mL, pooled mean (pooled SD)* | 0·51 (1·28) [N=221] | 0·45 (0·98) [N=141] | 0·62 (1·88) [N=50] | 0·58 (1·35) [N=30] | -0·421 (-0·94,0·098) | 0·112 | -0·317 (-0·882,0·249) | 0·272 |

Aggregate counts and summary statistics for the total sample and the MIS-C era subgroups were calculated for descriptive purposes only. Meta-analyses were computed by pooling risk differences (RD, categorical variables) or effect sizes (ES, continuous variables) and their 95% confidence intervals (CIs) previously calculated at site-level. A detailed definition of the variables based on EHR data or ICD-10 codes is reported as Supplementary Material.
*Centers had either normal or high sensitivity Troponin T available. Given difference in technologies, these have been pooled and analysed separately.

ALT: alanine transaminase; AST: aspartate transaminase; ES: effect size; N/L: neutrophil/lymphocyte; WBC: white blood count

**Supplemental Table 5.** Sensitivity analyses assessing demographic and clinical characteristics of patients with MIS-C according to SARS-CoV-2 variant eras (with cut off dates shifted by 2 weeks later).

| Variable | ***Delta* compared to A*lpha*** | | ***Omicron* compared to *Alpha*** | |
|---|---|---|---|---|
| | **Pooled RD or ES (95% CI)** | **P value** | **Pooled RD or ES (95% CI)** | **P value** |
| Age, years | -1·200 (-2·025, -0·375) | **0·004** | -2·032 (-3·025, -1·040) | **<0·001** |
| Sex* | 0·019 (-0·095, 0·118) | 0·745 | 0·051 (-0·197, 0·239) | 0·745 |
| Comorbidities | | | | |
| Overweight or obesity | -0·066 (-0·131,0·018) | 0·106 | -0·088 (-0·149,0·068) | 0·233 |
| Asthma | -0·042 (-0·121,0·036) | 0·465 | 0·065 (-0·083,0·182) | 0·776 |
| **Generalized or muco-cutaneous involvement** (other than fever) | 0·012 (-0·166,0·124) | 1·000 | -0·046 (-0·335,0·157) | 1·000 |
| Fatigue, asthenia | -0·012 (-0·102,0·088) | 1·000 | 0·013 (-0·153,0·131) | 1·000 |
| Rash | 0·048 (-0·136,0·172) | 0·715 | 0·023 (-0·106,0·154) | 1·000 |
| Conjunctivitis | 0·128 (-0·060,0·242) | 0·160 | 0·003 (-0·164,0·146) | 1·000 |
| Mucositis | 0·004 (-0·088,0·082) | 0·974 | -0·067 (-0·149,0·030) | 0·182 |
| Lymphadenitis/lymphadenopathy | -0·015 (-0·141,0·061) | 1·000 | 0·019 (-0·120,0·167) | 1·000 |
| **Gastrointestinal involvement** | 0·031 (-0·161,0·224) | 1·000 | 0·125 (-0·410,0·316) | 1·000 |
| Abdominal pain | -0·013 (-0·169,0·210) | 1·000 | 0·014 (-0·206,0·217) | 1·000 |
| Nausea or vomiting | -0·032 (-0·174,0·160) | 1·000 | 0·022 (-0·250,0·269) | 1·000 |
| Diarrhoea, enteritis, ileitis | -0·079 (-0·195,0·128) | 0·703 | -0·015 (-0·182,0·125) | 1·000 |
| Appendicitis, peritonitis | -0·002 (-0·029,0·043) | 1·000 | 0·041 (-0·015,0·135) | 0·490 |
| **Respiratory involvement** | -0·139 (-0·329, -0·026) | **0·016** | -0·164 (-0·415, 0·033) | 0·240 |
| Cough | -0·043 (-0·097,0·026) | 0·225 | -0·072 (-0·136,0·037) | 0·206 |
| Rhinitis/Rhinorrhoea | 0·000 (-0·038,0·061) | 1·000 | 0·017 (-0·052,0·109) | 1·000 |
| Sore throat | -0·043 (-0·101,0·026) | 0·302 | 0·003 (-0·138,0·123) | 1·000 |
| Respiratory failure/dyspnoea | -0·028 (-0·098,0·054) | 0·628 | -0·020 (-0·117,0·083) | 1·000 |
| Pleural effusion | -0·021 (-0·147,0·076) | 1·000 | -0·001 (-0·167,0·138) | 1·000 |
| Pulmonary oedema | -0·011 (-0·053,0·056) | 0·973 | 0·062 (-0·028,0·187) | 0·303 |
| Pneumonia | -0·133 (-0·265, -0·041) | **0·004** | -0·198 (-0·303,0·036) | 0·208 |
| ARDS | 0·002 (-0·041,0·054) | 1·000 | -0·025 (-0·070,0·054) | 0·523 |
| **Cardiovascular involvement** | -0·017 (-0·292,0·083) | 1·000 | -0·090 (-0·517,0·135) | 1·000 |
| Chest pain | -0·020 (-0·072,0·027) | 0·412 | -0·025 (-0·103,0·065) | 0·720 |
| Hypotension | -0·011 (-0·111,0·269) | 1·000 | 0·007 (-0·204,0·397) | 1·000 |
| Pre-syncope, syncope | 0·051 (-0·022,0·131) | 0·361 | -0·003 (-0·093,0·108) | 1·000 |
| Arrhythmias | -0·008 (-0·074,0·059) | 1·000 | -0·024 (-0·126,0·089) | 0·894 |
| Myocarditis | -0·068 (-0·230,0·030) | 0·195 | -0·084 (-0·302,0·054) | 0·272 |
| Pericarditis/pericardial effusion | 0·038 (-0·065,0·128) | 0·734 | -0·023 (-0·150,0·120) | 1·000 |
| Left ventricular dysfunction | -0·029 (-0·076,0·032) | 0·332 | -0·012 (-0·114,0·136) | 1·000 |
| Heart failure | -0·030 (-0·084,0·072) | 0·927 | -0·053 (-0·186,0·068) | 0·877 |
| Cardiogenic shock | -0·088 (-0·167,0·114) | 0·571 | 0·036 (-0·195,0·211) | 1·000 |
| **Shock (non-cardiogenic)/SIRS** | -0·328 (-0·515, -0·060) | **0·007** | -0·110 (-0·322,0·030) | 0·138 |
| Septic shock | -0·117 (-0·177,0·010) | 0·067 | -0·009 (-0·089,0·076) | 1·000 |
| Hypovolemic shock | -0·005 (-0·037,0·052) | 1·000 | 0·050 (-0·029,0·151) | 0·338 |
| Shock, others (non-cardiogenic) | -0·070 (-0·178,0·087) | 0·479 | 0·011 (-0·140,0·180) | 1·000 |
| SIRS | -0·276 (-0·431, -0·022) | **0·009** | -0·180 (-0·340, -0·047) | **0·006** |
| **Neurologic involvement** | -0·126 (-0·336,0·063) | 0·448 | -0·074 (-0·314,0·084) | 0·500 |
| Headache | -0·052 (-0·166,0·049) | 1·000 | -0·030 (-0·188,0·094) | 1·000 |
| Disorientation/Confusion | -0·044 (-0·118,0·033) | 0·359 | -0·042 (-0·108,0·056) | 0·515 |
| Seizures | 0·012 (-0·034,0·059) | 0·932 | -0·022 (-0·071,0·054) | 0·536 |
| Muscle weakness/myalgia/myositis | 0·018 (-0·086,0·119) | 1·000 | 0·039 (-0·096,0·115) | 0·799 |
| Encephalopathy/meningoencephalitis | -0·057 (-0·220,0·065) | 0·739 | 0·004 (-0·095,0·063) | 1·000 |
| Stroke | 0·000 (-0·029,0·042) | 1·000 | 0·019 (-0·038,0·102) | 0·881 |
| **Renal dysfunction** | 0·009 (-0·252,0·117) | 1·000 | -0·020 (-0·236,0·106) | 1·000 |
| **Liver dysfunction** | -0·009 (-0·081,0·078) | 1·000 | 0·034 (-0·098,0·218) | 1·000 |

*Male as reference category.
Meta-analyses were computed by pooling risk differences (RD, categorical variables) or effect sizes (ES, continuous variables) and their 95% confidence intervals (CIs) previously calculated at site-level. A detailed definition of the variables based on EHR data or ICD-10 codes is reported as Supplementary Material. A detailed definition of the variables based on EHR data or ICD-10 codes is reported as Supplementary Material.
Total N=436; Alpha N=277, Delta N=99, Omicron N=60. For age only (including CHOP): N=598; Alpha N=385, Delta N=132, Omicron N=81.
ES: effect size; MIS-C: multisystem inflammatory syndrome; RD: risk difference; SD: standard deviation; SIRS: systemic inflammatory response syndrome.

**Supplemental Table 6.** Sensitivity analyses assessing patient-level outcomes in patients with MIS-C according to SARS-CoV-2 eras (with cut off dates shifted by 2 weeks later).

| **Variable** | ***Delta* compared to *Alpha*** | | ***Omicron* compared to *Alpha*** | |
|---|---|---|---|---|
| | **Pooled RD or ES (95% CI)** | **P value** | **Pooled RD or ES (95% CI)** | **P value** |
| ICU admission | -0·025 (-0·123, 0·038) | 0·477 | 0·027 (-0·286, 0·174) | 1·000 |
| Diuretic therapy | -0·052 (-0·147, 0·018) | 0·155 | 0·110 (-0·052, 0·304) | 0·409 |
| Anticoagulation therapy | 0·002 (-0·237, 0·080) | 1·000 | -0·258 (-0·490, -0·095) | **0·001** |
| Sedation or muscle relaxant | -0·036 (-0·150, 0·026) | 0·268 | -0·011 (-0·132, 0·074) | 0·985 |
| Vasoactive/inotropic support | -0·009 (-0·046, 0·070) | 1·000 | 0·054 (-0·163, 0·065) | 0·367 |
| O2 supplementation or MV | 0·014(-0·041,0·084) | 0·992 | -0·029(-0·131,0·078) | 1·000 |
| Cardiac arrest | -0·007 (-0·036, 0·035) | 0·788 | -0·007 (-0·050, 0·067) | 1·000 |
| ECMO | -0·002 (-0·031, 0·040) | 1·000 | -0·007 (-0·047, 0·070) | 1·000 |
| Coronary aneurysm | -0·011 (-0·062,0·098) | 1·000 | 0·079 (-0·051,0·222) | 0·580 |
| Coronary artery thrombosis or myocardial infarct | -0·009 (-0·036,0·036) | 0·753 | -0·010 (-0·049,0·069) | 0·926 |
| Composite adverse cardiovascular outcome* | -0·059 (-0·206, 0·095) | 0·515 | -0·001 (-0·231, 0·151) | 1·000 |
| Length of hospitalization, days | -1·416 (-2·285, 0·546) | **0·001** | -1·035 (-2·171, 0·100) | 0·074 |

Meta-analyses were computed by pooling risk differences (RD, categorical variables) or effect sizes (ES, continuous variables) and their 95% confidence intervals (CIs) previously calculated at site-level. A detailed definition of the variables based on EHR data or ICD-10 codes is reported as Supplementary Material.
*Composite cardiovascular outcome: ventricular dysfunction, heart failure or cardiogenic shock (ICD-10 codes), inotropic/vasoactive drugs (EHR data), coronary aneurysm (ICD-10 codes), major arrhythmias (ICD-10 codes) cardiac arrest (EHR data and ICD-codes), VA-ECMO (EHR data).

Total N=436; Alpha N=277, Delta N=99, Omicron N=60. For length of hospitalization only (including CHOP): N=598; Alpha N=385, Delta N=132, Omicron N=81.
ECMO: Extracorporeal Membrane Oxygenation; ES: effect size; ICU: intensive care unit; MIS-C: multisystem inflammatory syndrome; MV: mechanical ventilation; O2: oxygen; RD: risk difference; SD: standard deviation.

**Supplemental Table 7.** Sensitivity analyses assessing laboratory values at admission and worst during hospitalization in patients with MIS-C according to SARS-CoV-2 eras (with cut off dates shifted by 2 weeks later).

| Variable | ***Delta* compared to *Alpha*** | | ***Omicron* compared to *Alpha*** | |
|---|---|---|---|---|
| | **Pooled ES (95% CI)** | **P value** | **Pooled ES (95% CI)** | **P value** |
| **At admission** | | | | |
| WBC, x10$^9$/uL | 0·091 (-1·512, 1·694) | 0·911 | -1·791 (-3·488, -0·093) | **0·039** |
| Lymphocyte count, x10$^9$/uL | -0·152 (-0·293, -0·011) | **0·035** | -0·378 (-0·521, -0·235) | **<0·001** |
| Neutrophil count, x10$^9$/uL | 0·801 (-0·274, 1·876) | 0·144 | -1·021 (-2·007, -0·034) | 0·043 |
| N/L ratio | 1·1 (-0·5, 2·8) | 0·188 | 0·8 (-1·6, 3·2) | 0·518 |
| Platelets count, x10$^9$/uL | 3·532 (-12·764, 19·827) | 0·671 | -7·372 (-25·187, 10·443) | 0·417 |
| C-reactive protein, mg/L | -27·8 (-44·5, -11·1) | **0·001** | 0·6 (-23·6, 24·8) | 0·960 |
| Albumin, g/dL | 0·1 (0·01, 0·2) | **0·027** | 0·2 (0·04, 0·3) | **0·008** |
| D-dimer, ng/mL | -364·8 (-1138·5, 408·8) | 0·355 | -884·4 (-1747·3, -21·4) | **0·045** |
| Prothrombin time, s | -0·4 (-0·8, -0·01) | **0·046** | -0·1 (-0·7, 0·5) | 0·749 |
| Fibrinogen, mg/dL | -15·2 (-46·5, 16·0) | 0·339 | 21·4 (-22·6, 65·4) | 0·341 |
| Ferritin, ug/L | 113·3 (-74·3, 300·9) | 0·236 | 82·9 (-114·3, 280·1) | 0·410 |
| ALT, U/L | -3·5 (-10·4, 3·5) | 0·332 | 1·5 (-8·6, 11·5) | 0·771 |
| AST, U/L | -8·0 (-19·2, 3·2) | 0·16 | -7·5 (-20·1, 5·0) | 0·240 |
| Creatinine, mg/dL | -0·06 (-0·14, 0·02) | 0·117 | -0·06 (-0·16, 0·04) | 0·264 |
| Troponin T, normal sensitivity, ng/mL | -0·13 (-0·24, -0·03) | **0·012** | -0·11 (-0·24, 0·02) | 0·105 |
| Troponin T, high sensitivity, ng/mL | -0·17 (-0·59, 0·25) | 0·420 | -0·06 (-0·66, 0·53) | 0·837 |
| **During hospitalization** | | | | |
| WBC, highest, x10$^9$/uL | -0·526 (-2·266, 1·215) | 0·554 | -0·689 (-3·33, 1·953) | 0·609 |
| Lymphocyte count, lowest, x10$^9$/uL | -0·092 (-0·208, 0·024) | 0·119 | -0·3 (-0·433, -0·168) | **<0·001** |
| Neutrophil count, highest, x10$^9$/uL | 0·873 (-0·350, 2·096) | 0·162 | 0·228 (-1·205, 1·662) | 0·755 |
| N/L ratio, highest | 1·0 (-0·7, 2·7) | 0·258 | 2·0 (-0·5, 4·5) | 0·119 |
| Platelets count, highest, x10$^9$/uL, | -13·369 (-41·393, 14·654) | 0·35 | -11·714 (-49·860, 26·431) | 0·547 |
| C-reactive protein, highest, mg/L | -12·3 (-27·8 , 3·3) | 0·122 | -8·3 (-27·5, 10·8) | 0·393 |
| Albumin, lowest, g/dL | -0·01 (-0·08, 0·08) | 0·985 | -0·08 (-0·18, 0·01) | 0·090 |
| D-dimer, highest, ng/mL | -434·7 (-1197·9, 328·6) | 0·264 | -608·9 (-1400·8, 183·1) | 0·132 |
| Prothrombin time, highest, s | -0·5 (-0·9,-0·1) | **0·008** | -0·3 (-1·0, 0·3) | 0·336 |
| Fibrinogen, highest, mg/dL | -2·549(-34·341,29·243) | 0·875 | 4·68(-37·327,46·686) | 0·827 |
| Ferritin, highest, ug/L | 84·0 (-131·2, 299·1) | 0·444 | 148·4 (-128·4, 425·2) | 0·293 |
| ALT, highest, U/L | -9·8 (-21·0, 1·3) | 0·085 | 0·9 (-15·3, 17·1) | 0·911 |
| AST, highest, U/L | -7·6 (-19·3, 4·1) | 0·202 | -5·5 (-20·6, 9·5) | 0·470 |
| Creatinine, highest, mg/dL | -0·05 (-0·11 ,0·01) | 0·143 | 0·01 (-0·07, 0·09) | 0·901 |
| Troponin-T, normal sensitivity, highest, ng/mL* | -0·16 (-0·28, -0·03) | **0·012** | -0·149 (-0·288, -0·01) | **0·035** |
| Troponin-T, high sensitivity, highest, ng/mL* | -0·39 (-0·88 ,0·11) | 0·126 | -0·34 (-0·99 ,0·30) | 0·300 |

Aggregate counts and summary statistics for the total sample and the MIS-C era subgroups were calculated for descriptive purposes only. Meta-analyses were computed by pooling risk differences (RD, categorical variables) or effect sizes (ES, continuous variables) and their 95% confidence intervals (CIs) previously calculated at site-level. A detailed definition of the variables based on EHR data or ICD-10 codes is reported as Supplementary Material.
*Centers had either normal or high sensitivity Troponin-T available. Given difference in technologies, these have been pooled and analysed separately.

Total N=598; Alpha N=385, Delta N=132, Omicron N=81.
ALT: alanine transaminase; AST: aspartate transaminase; ES: effect size; N/L: neutrophil/lymphocyte; WBC: white blood count

**Supplemental Table 8.** Laboratory test details, including units, Logical Observation Identifier Names and Codes (LOINC) code, and definition for worst value.

| Laboratory Test | Units | LOINC Code | Worst value definition |
|---|---|---|---|
| White blood cell count (Leukocytes) | 10*3/uL | 6690-2 | highest |
| Lymphocyte | 10*3/uL | 731-0 | lowest |
| Neutrophil | 10*3/uL | 751-8 | highest |
| Platelets | 10*3/uL | 777-3 | highest |
| Albumin | g/dL | 1751-7 | lowest |
| D-dimer | ng/mL | 48065-7,<br>48066-5 | highest |
| Prothrombin time (PT) | s | 5902-2 | highest |
| Fibrinogen | mg/dL | 3255-7 | highest |
| Ferritin | ng/mL | 2276-4 | highest |
| Alanine aminotransferase (ALT) | U/L | 1742-6 | highest |
| Aspartate aminotransferase (AST) | U/L | 1920-8 | highest |
| Creatinine | mg/dL | 2160-0 | highest |
| Troponin-T normal sensitivity | ng/mL | 6598-7 | highest |
| Troponin-T high sensitivity | ng/mL | 49563-0 | highest |
| C-reactive protein | mg/L | 1988-5 | highest |

Abbreviations: LOINC: Logical Observation Identifier Names and Codes

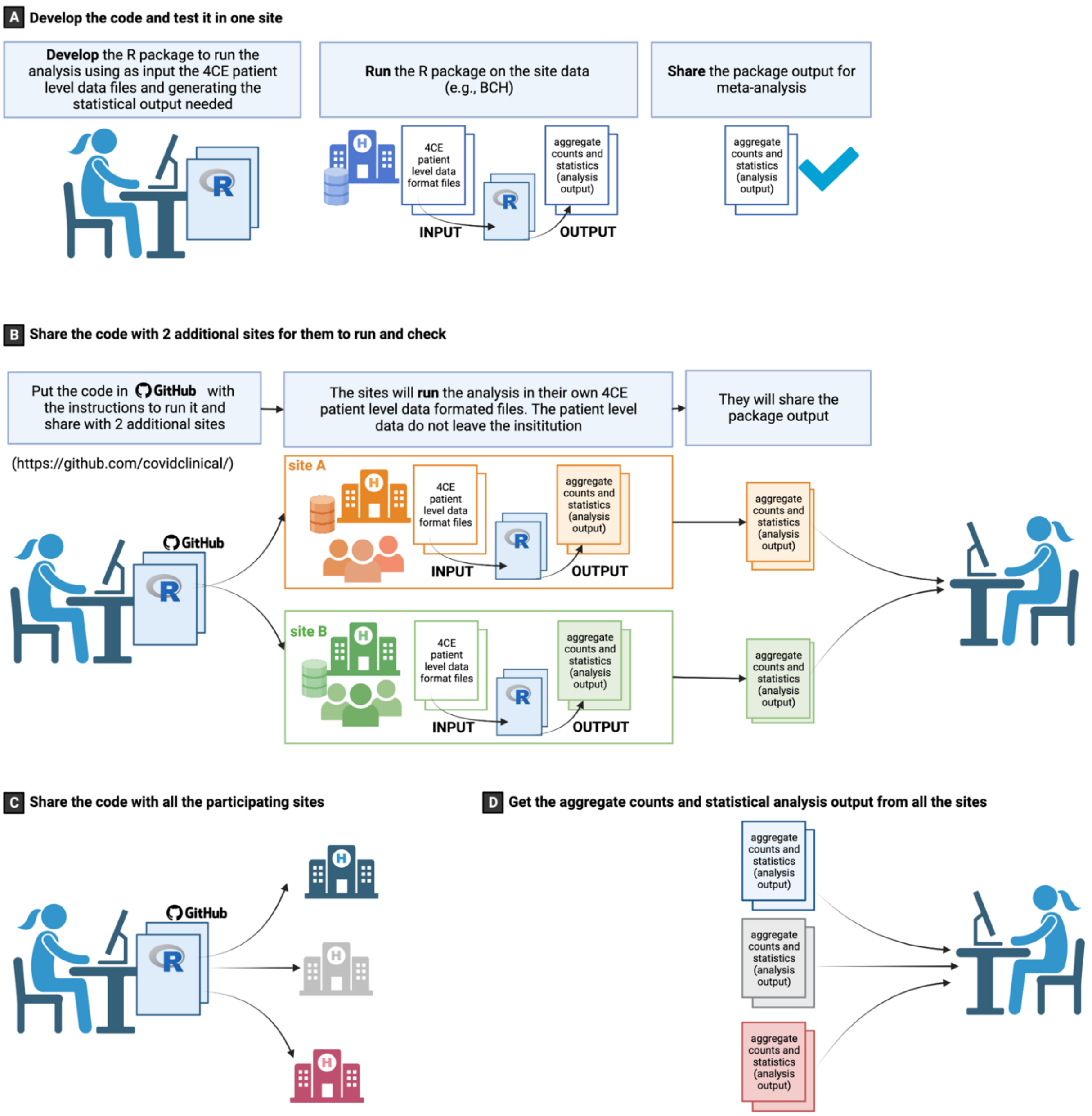


**Supplemental Figure 1**. Schematics of the 4CE Consortium federated approach to develop and conduct analyses across sites.

4CE: The Consortium for Clinical Characterization of COVID-19 by EHR

**Supplemental Methods 1**. Data quality check procedure.

*Data quality control procedures*

Quality control procedures were performed at both site-level and across sites. The initial site-level quality control included: careful check of the study period dates, total patient counts, procedures counts, medication counts, as well as summary statistics of the lab values (min, max, mean, standard deviation) to check for extraction errors or outliers. Outliers were identified and investigated, and modifications were made to the data extraction as necessary (e.g., extreme lab values and outliers in the length of hospitalization). When needed, in case of outliers, patient inclusion criteria were reviewed and re-discussed with the site MIS-C experts. Additionally, any potentially missing data for specific timepoints or values were reviewed with the data analysis team at each site.

Aggregate data were subsequently checked and compared across all sites by two experts in bioinformatics (A.G., S.M.) and by an expert in pediatric cardiology and critical care (F.S.). To facilitate this process, we developed a dedicated advanced visualization tool using R Shiny App (R Studio, R Foundation for Statistical Computing, Vienna, Austria). The app is publicly available at https://avillachlab.shinyapps.io/a1l1non3/. The app compares ICD-10-code type and counts and percentages, ICD-10-code based categories counts, as well as summary statistics of age, laboratory data, and length of hospitalization among centers. ICD-10-code type and counts were extensively reviewed to refine the list of ICD-10 codes included for the analysis. This was particularly useful given the significant heterogeneity existing within U.S. and European codes. ICD-10-code based categories counts and percentages were compared among centers to identify any potential patterns. Laboratory values and patient percentages were also compared across sites to identify potential discrepancies in units and/or missing data. Any incongruence or missing data were discussed with the sites and among the group of leading authors when a major decision was requested. Given that CHOP ICD-10-code based data did not pass the quality check control for ICD-10-codes data, these data were excluded from analysis.

**Supplemental Methods 2.** List of ICD-10 codes of interest.

**Demographics of interest**

**Obesity:** E236, E278, E6601, E662, E6609, E661, E668, E669, E68
BMI≥30: Z6830-Z685; BMI pediatric≥95%ile for age: Z6854

**Overweight:** E66.3, E6693
BMI≥25 to 30: Z6825-Z6829; BMI pediatric≥85% and <95% for age: Z6853

**Generalized and cutaneous symptoms:**

**Fatigue (acute)/ asthenia:** R53, R531, R5381, R5382, R5383,
**Rash/erythema:** R21, L539, L519, L538, L509
**Conjunctivitis:** H10.9, H103, H1030, H1031, H1033, H1089, H108, H118, H100, H11433, H11439
**Lymphadenitis/Lymphadenopathy:** L040, L048, L049, R590, R591, R599, R221
**Dehydration:** E860, E86, E861, E869
**Fluid overload/generalized oedema:** E877, E8770, R601, R609, E8779

**Gastrointestinal symptoms:**

**Abdominal pain:** R100, R101, R102, R109, R1010, R1011, R1012, R1013, R103, R1030, R1031, R1032, R1033, R1083, R1084, R104, R10812, R10813, R10814, R10817, R10819, R10823
**Nausea/ Vomiting:** R11, R110, R112, R1110, R1111, R1112, R1114, R112
**Diarrhoea/Enteritis/Ileitis/Ileus:** R197, K580, K589, K582, P783, K529, K589, A099, K567
**Appendicitis/Peritonitis:** K3530, K3531, K3533, K3580, K36, K37, K65.9
**Hepato-splenomegaly**: R160, R161, R162
**Ascites:** R18, R188

**Upper airway/ Respiratory symptoms:**

**Mucositis:** K123, K1230, K1239, K130, K137
**Cough:** R05, R051, R052, R053, R058, R059
**Rhinitis/Rhinorrhoea/upper respiratory infection:** J00, J069, R0981, R0982
**Sore throat (acute):** J029, R070
**Pneumonia/bronchitis/lower respiratory infection:** J1281, J1289, J181, J189, J208, J22
**Dyspnoea/Shortness of breath:** R060, R0600, R0601, R0602, R0603
**Respiratory failure:** J9600, J9601, J9602, J9620, J9621, J9622, J9690, J9691, J9692, J9609, J9699
**Acute respiratory distress syndrome:** J80
**Pulmonary oedema:** J81, J810
**Pleural effusion:** J90, J91

**Neurologic symptoms:**

**Headache:** R51, R519, G43909, G44009, G44209, G44219, G4453, G4484, G4485, G43909, G439
**Disorientation/Confusion:** R410, R418, R4182
**Dizziness:** R42
**Encephalopathy (acute)/ Meningoencephalitis:** G96.9, G934, G9340, G048, G049, G0490, G9349, G039
**Seizures:** R569, G40909, G40419, G40802, G40804, G4089, G40909, G40919
**Stroke (ischemic)/transient cerebral ischemic attack:** I639, I6340, I63412, I6349, I6350, I6359, I6782, G459
Cerebral haemorrhages: 1619, I629
**Muscle weakness/myalgia/myositis:** M6281, M609, M628, M6280, M6282, M791, M7910, M7916, M7919
**Ataxia/gait and mobility abnormalities:** R270, R268, R2689, R278
**Polyneuropathy:** G629

**Cardiovascular symptoms:**

**Chest pain:** R071, R072, R703, R704, R0781, R0789, R079
**Palpitations:** R002
**Hypotension**: I959, I958, I9589
**Pre-syncope, syncope:** R55, R054
**Left Ventricular dysfunction**: I5010, I5011, I5012, I501
**Heart failure:** I5089, I509, plus
Systolic: I5020, I5021, I5023, I50.82, I50.9,
Diastolic: I5030, I5031, I5033,
Unspecified or combined: I500, I5000, I5040, I5041, I5043
Right HF: I50810, I50811, I50813, I50814

**Mitral valve regurgitation:** I340
**Arrhythmias: Major:** AV block, second degree: I440, AV block, complete: I442, Supraventricular tachycardia: I471, Atrial fibrillation/flutter: I48, Ventricular arrhythmias: I470, I472
**Minor/others:** Sick sinus syndrome: 1495, AV block, first degree: I440, Other conduction disorders: I4589, I459, Long QT: I4581, Others: I498, I499
**Shock, cardiogenic:** R570, R579
**Pulmonary embolism:** I2699
**Coronary aneurysm:** I2541, I254
**Coronary thrombosis or myocardial infarction:** I209, 219, I240, I249
**Pericarditis, pericardial effusion:** I300, I308, I309, I313
**Myocarditis:** I400, I408, I409, I41, I411, I418, I514
**Cardiac arrest:** I460, I462, I469

<u>**SIRS or non-cardiogenic shock**</u>
**Hypovolemic shock:** R571
**Septic, shock:** R572, R6521
**Unspecified/other shock:** R578, R579
**Systemic inflammatory response syndrome:** R650, R651, R6510, R6511, R652, R653, R659

<u>**Renal involvement**</u>
**Renal dysfunction or failure, acute:** N170, N171, N172, N179, N178, N19, R944

<u>**Liver involvement:**</u>
**Liver dysfunction or failure, acute:** R7401, K7689, K769, K7200, K729, K7290, R945

<u>**Laboratory profile**</u>
**Abnormal white blood count:** Elevated WBC D72829**,** Decreased WBC D72819**,** Lymphocytopenia D72810**,** Others D728, D7289
**Anaemia:** D508, D509, D649, D500
**Thrombocytopenia:** D696
**Coagulation abnormalities:** D89, D688, R791, D65, D684, D689, D686, D6869
**Electrolytes abnormalities:** E833, E8330, E8339, E834, E8341, E8342, E835, E8351, E8358, E870, E871, E875, E8758, E876, E8760, E8768, Hyponatremia: E871, E8718, Hypokalaemia: E876, E8760, E8768
**Hyperglycaemia:** R739
**Acidosis:** E872
**Alkalosis:** E873
**Elevated lactated dehydrogenases:** R740

Source: https://icd10cmtool.cdc.gov

**Supplemental Methods 3.** List of EHR-based 4CE data definitions.

**Procedures**

Oxygen supplementation or mechanical ventilation (MV):
- Oxygen therapy only
- Non-invasive ventilation
- Continuous positive airway pressure (CPAP) MV
- Endotracheal intubation with invasive MV

Extracorporeal membrane oxygenation (ECMO):
- Veno-venous (VV) ECMO
- Veno-arterial (VA) ECMO

Cardiopulmonary resuscitation (CPR)

**Medications**

Diuretic therapy: furosemide, bumetanide, torsemide, piretanide, ethacrynic acid

Anticoagulation therapy: warfarin, heparin, enoxaparin, dalteparin, fondaparinux, bivalirudin, tirofiban, apixaban

Sedation or muscle relaxant: propofol, dexmedetomidine, midazolam, opioids, ketamine, vecuronium, rocuronium, cisatracurium

Inotropic/vasoactive support: epinephrine, norepinephrine, dopamine, dobutamine, phenylephrine, vasopressin, milrinone

**Supplemental Information 1. The Consortium for Clinical Characterization of COVID-9 by EHR (4CE) Members**

| First Name | Last Name |
|---|---|
| James R | Aaron |
| Atif | Adam |
| Giuseppe | Agapito |
| Adem | Albayrak |
| Giuseppe | Albi |
| Mario | Alessiani |
| Anna | Alloni |
| Danilo F | Amendola |
| François | Angoulvant |
| Li L L J | Anthony |
| Bruce J | Aronow |
| Fatima | Ashraf |
| Andrew | Atz |
| Paul | Avillach |
| Vidul | Ayakulangara Panickan |
| Paula S | Azevedo |
| Rafael | Badenes |
| James | Balshi |
| Ashley | Batugo |
| Brendin R | Beaulieu-Jones |
| Brett K | Beaulieu-Jones |
| Douglas S | Bell |
| Antonio | Bellasi |
| Riccardo | Bellazzi |
| Vincent | Benoit |
| Michele | Beraghi |
| José Luis | Bernal-Sobrino |
| Mélodie | Bernaux |
| Romain | Bey |
| Surbhi | Bhatnagar |
| Alvar | Blanco-Martínez |
| Martin | Boeker |
| Clara-Lea | Bonzel |
| John | Booth |
| Silvano | Bosari |
| Florence T | Bourgeois |
| Robert L | Bradford |
| Gabriel A | Brat |
| Stéphane | Bréant |
| Nicholas W | Brown |
| Raffaele | Bruno |
| William A | Bryant |
| Mauro | Bucalo |
| Emily | Bucholz |
| Anita | Burgun |
| Tianxi | Cai |
| Mario | Cannataro |
| Aldo | Carmona |
| Anna Maria | Cattelan |
| Charlotte | Caucheteux |
| Julien | Champ |
| Jin | Chen |
| Krista Y | Chen |

| | |
|---|---|
| Luca | Chiovato |
| Lorenzo | Chiudinelli |
| Kelly | Cho |
| James J | Cimino |
| Tiago K | Colicchio |
| Sylvie | Cormont |
| Sébastien | Cossin |
| Jean B | Craig |
| Juan Luis | Cruz-Bermúdez |
| Jaime | Cruz-Rojo |
| Arianna | Dagliati |
| Mohamad | Daniar |
| Christel | Daniel |
| Priyam | Das |
| Batsal | Devkota |
| Audrey | Dionne |
| Rui | Duan |
| Julien | Dubiel |
| Scott L | DuVall |
| Loic | Esteve |
| Hossein | Estiri |
| Shirley | Fan |
| Robert W | Follett |
| Thomas | Ganslandt |
| Noelia | García-Barrio |
| Lana X | Garmire |
| Nils | Gehlenborg |
| Emily J | Getzen |
| Alon | Geva |
| Rachel SJ | Goh |
| Tomás | González González |
| Tobias | Gradinger |
| Alexandre | Gramfort |
| Romain | Griffier |
| Nicolas | Griffon |
| Olivier | Grisel |
| Alba | Gutiérrez-Sacristán |
| Pietro H | Guzzi |
| Larry | Han |
| David A | Hanauer |
| Christian | Haverkamp |
| Derek Y | Hazard |
| Bing | He |
| Darren W | Henderson |
| Martin | Hilka |
| Yuk-Lam | Ho |
| John H | Holmes |
| Jacqueline P | Honerlaw |
| Chuan | Hong |
| Kenneth M | Huling |
| Meghan R | Hutch |
| Richard W | Issitt |
| Anne Sophie | Jannot |
| Vianney | Jouhet |
| Mundeep K | Kainth |
| Kernan F | Kate |
| Ramakanth | Kavuluru |
| Mark S | Keller |

| | |
|---|---|
| Chris J | Kennedy |
| Kate F | Kernan |
| Daniel A | Key |
| Katie | Kirchoff |
| Jeffrey G | Klann |
| Isaac S | Kohane |
| Ian D | Krantz |
| Detlef | Kraska |
| Ashok K | Krishnamurthy |
| Sehi | L'Yi |
| Judith | Leblanc |
| Guillaume | Lemaitre |
| Leslie | Lenert |
| Damien | Leprovost |
| Molei | Liu |
| Ne Hooi Will | Loh |
| Qi | Long |
| Sara | Lozano-Zahonero |
| Yuan | Luo |
| Kristine E | Lynch |
| Sadiqa | Mahmood |
| Sarah E | Maidlow |
| Adeline | Makoudjou |
| Simran | Makwana |
| Alberto | Malovini |
| Kenneth D | Mandl |
| Chengsheng | Mao |
| Anupama | Maram |
| Monika | Maripuri |
| Patricia | Martel |
| Marcelo R | Martins |
| Jayson S | Marwaha |
| Aaron J | Masino |
| Maria | Mazzitelli |
| Diego R | Mazzotti |
| Arthur | Mensch |
| Marianna | Milano |
| Marcos F | Minicucci |
| Bertrand | Moal |
| Taha | Mohseni Ahooyi |
| Jason H | Moore |
| Cinta | Moraleda |
| Jeffrey S | Morris |
| Michele | Morris |
| Karyn L | Moshal |
| Sajad | Mousavi |
| Danielle L | Mowery |
| Douglas A | Murad |
| Shawn N | Murphy |
| Thomas P | Naughton |
| Carlos Tadeu Breda | Neto |
| Antoine | Neuraz |
| Jane | Newburger |
| Kee Yuan | Ngiam |
| Wanjiku FM | Njoroge |
| James B | Norman |
| Jihad | Obeid |
| Marina P | Okoshi |

| | |
|---|---|
| Karen L | Olson |
| Gilbert S. | Omenn |
| Nina | Orlova |
| Brian D | Ostasiewski |
| Nathan P | Palmer |
| Nicolas | Paris |
| Lav P | Patel |
| Miguel | Pedrera-Jiménez |
| Ashley C | Pfaff |
| Emily R | Pfaff |
| Danielle | Pillion |
| Sara | Pizzimenti |
| Tanu | Priya |
| Hans U | Prokosch |
| Robson A | Prudente |
| Andrea | Prunotto |
| Víctor | Quirós-González |
| Rachel B | Ramoni |
| Maryna | Raskin |
| Siegbert | Rieg |
| Gustavo | Roig-Domínguez |
| Pablo | Rojo |
| Nekane | Romero-Garcia |
| Paula | Rubio-Mayo |
| Paolo | Sacchi |
| Carlos | Sáez |
| Elisa | Salamanca |
| Malarkodi Jebathilagam | Samayamuthu |
| L. Nelson | Sanchez-Pinto |
| Arnaud | Sandrin |
| Nandhini | Santhanam |
| Janaina C.C | Santos |
| Fernando J | Sanz Vidorreta |
| Maria | Savino |
| Emily R | Schriver |
| Petra | Schubert |
| Juergen | Schuettler |
| Luigia | Scudeller |
| Neil J | Sebire |
| Pablo | Serrano-Balazote |
| Patricia | Serre |
| Arnaud | Serret-Larmande |
| Mohsin A | Shah |
| Zahra | Shakeri Hossein Abad |
| Domenick | Silvio |
| Piotr | Sliz |
| Jiyeon | Son |
| Charles | Sonday |
| Andrew M | South |
| Francesca | Sperotto |
| Anastasia | Spiridou |
| Zachary H. | Strasser |
| Amelia LM | Tan |
| Bryce W.Q. | Tan |
| Byorn W.L. | Tan |
| Suzana E | Tanni |
| Deanne M | Taylor |
| Ana I | Terriza-Torres |

| | |
|---|---|
| Valentina | Tibollo |
| Patric | Tippmann |
| Emma MS | Toh |
| Carlo | Torti |
| Enrico M | Trecarichi |
| Andrew K | Vallejos |
| Gael | Varoquaux |
| Margaret E | Vella |
| Guillaume | Verdy |
| Jill-Jênn | Vie |
| Shyam | Visweswaran |
| Michele | Vitacca |
| Kavishwar B | Wagholikar |
| Lemuel R | Waitman |
| Xuan | Wang |
| Demian | Wassermann |
| Griffin M | Weber |
| Martin | Wolkewitz |
| Scott | Wong |
| Zongqi | Xia |
| Xin | Xiong |
| Ye | Ye |
| Nadir | Yehya |
| William | Yuan |
| Joany M | Zachariasse |
| Janet J | Zahner |
| Alberto | Zambelli |
| Harrison G | Zhang |
| Daniela | Zöller |
| Valentina | Zuccaro |
| Chiara | Zucco |
