# Supplementary material for: Clinical phenotypes and outcomes in children with multisystem inflammatory syndrome across SARS-CoV-2 variant eras: a multinational study from the 4CE consortium
Source: eClinicalMedicine. 2023 Sep 14;64:102212. doi: 10.1016/j.eclinm.2023.102212 (PMC10511777; doi:10.1016/j.eclinm.2023.102212)
Supplement: Members List [file mmc2.pdf]

The Consortium for Clinical Characterization of COVID-9 by EHR (4CE) Members

First Name	Last Name
James R	Aaron
Atif	Adam
Giuseppe	Agapito
Adem	Albayrak
Giuseppe	Albi
Mario	Alessiani
Anna	Alloni
Danilo F	Amendola
François	Angoulvant
Li L L J	Anthony
Bruce J	Aronow
Fatima	Ashraf
Andrew	Atz
Paul	Avillach
Vidul	Ayakulangara Panickan
Paula S	Azevedo
Rafael	Badenes
James	Balshi
Ashley	Batugo
Brendin R	Beaulieu-Jones
Brett K	Beaulieu-Jones
Douglas S	Bell
Antonio	Bellasi
Riccardo	Bellazzi
Vincent	Benoit
Michele	Beraghi
José Luis	Bernal-Sobrino
Mélodie	Bernaux
Romain	Bey
Surbhi	Bhatnagar
Alvar	Blanco-Martínez
Martin	Boeker
Clara-Lea	Bonzel
John	Booth
Silvano	Bosari
Florence T	Bourgeois
Robert L	Bradford
Gabriel A	Brat
Stéphane	Bréant
Nicholas W	Brown
Raffaele	Bruno
William A	Bryant
Mauro	Bucalo
Emily	Bucholz
Anita	Burgun
Tianxi	Cai
Mario	Cannataro
Aldo	Carmona
Anna Maria	Cattelan
Charlotte	Caucheteux
Julien	Champ
Jin	Chen

Krista Y	Chen
Luca	Chiovato
Lorenzo	Chiudinelli
Kelly	Cho
James J	Cimino
Tiago K	Colicchio
Sylvie	Cormont
Sébastien	Cossin
Jean B	Craig
Juan Luis	Cruz-Bermúdez
Jaime	Cruz-Rojo
Arianna	Dagliati
Mohamad	Daniar
Christel	Daniel
Priyam	Das
Batsal	Devkota
Audrey	Dionne
Rui	Duan
Julien	Dubiel
Scott L	DuVall
Loic	Esteve
Hossein	Estiri
Shirley	Fan
Robert W	Follett
Thomas	Ganslandt
Noelia	García-Barrio
Lana X	Garmire
Nils	Gehlenborg
Emily J	Getzen
Alon	Geva
Rachel SJ	Goh
Tomás	González González
Tobias	Gradinger
Alexandre	Gramfort
Romain	Griffier
Nicolas	Griffon
Olivier	Grisel
Alba	Gutiérrez-Sacristán
Pietro H	Guzzi
Larry	Han
David A	Hanauer
Christian	Haverkamp
Derek Y	Hazard
Bing	He
Darren W	Henderson
Martin	Hilka
Yuk-Lam	Ho
John H	Holmes
Jacqueline P	Honerlaw
Chuan	Hong
Kenneth M	Huling
Meghan R	Hutch
Richard W	Issitt
Anne Sophie	Jannot
Vianney	Jouhet
Mundeep K	Kainth

Ramakanth	Kavuluru
Mark S	Keller
Chris J	Kennedy
Kate F	Kernan
Daniel A	Key
Katie	Kirchoff
Jeffrey G	Klann
Isaac S	Kohane
Ian D	Krantz
Detlef	Kraska
Ashok K	Krishnamurthy
Sehi	L'Yi
Judith	Leblanc
Guillaume	Lemaitre
Leslie	Lenert
Damien	Leprovost
Molei	Liu
Ne Hooi Will	Loh
Qi	Long
Sara	Lozano-Zahonero
Yuan	Luo
Kristine E	Lynch
Sadiqa	Mahmood
Sarah E	Maidlow
Adeline	Makoudjou
Simran	Makwana
Alberto	Malovini
Kenneth D	Mandl
Chengsheng	Mao
Anupama	Maram
Monika	Maripuri
Patricia	Martel
Marcelo R	Martins
Jayson S	Marwaha
Aaron J	Masino
Maria	Mazzitelli
Diego R	Mazzotti
Arthur	Mensch
Marianna	Milano
Marcos F	Minicucci
Bertrand	Moal
Taha	Mohseni Ahooyi
Jason H	Moore
Cinta	Moraleda
Jeffrey S	Morris
Michele	Morris
Karyn L	Moshal
Sajad	Mousavi
Danielle L	Mowery
Douglas A	Murad
Shawn N	Murphy
Thomas P	Naughton
Carlos Tadeu Breda	Neto
Antoine	Neuraz
Jane	Newburger
Kee Yuan	Ngiam

Wanjiku FM	Njoroge
James B	Norman
Jihad	Obeid
Marina P	Okoshi
Karen L	Olson
Gilbert S.	Omenn
Nina	Orlova
Brian D	Ostasiewski
Nathan P	Palmer
Nicolas	Paris
Lav P	Patel
Miguel	Pedrera-Jiménez
Ashley C	Pfaff
Emily R	Pfaff
Danielle	Pillion
Sara	Pizzimenti
Tanu	Priya
Hans U	Prokosch
Robson A	Prudente
Andrea	Prunotto
Víctor	Quirós-González
Rachel B	Ramoni
Maryna	Raskin
Siegbert	Rieg
Gustavo	Roig-Domínguez
Pablo	Rojo
Nekane	Romero-Garcia
Paula	Rubio-Mayo
Paolo	Sacchi
Carlos	Sáez
Elisa	Salamanca
Malarkodi Jebathilagam	Samayamuthu
L. Nelson	Sanchez-Pinto
Arnaud	Sandrin
Nandhini	Santhanam
Janaina C.C	Santos
Fernando J	Sanz Vidorreta
Maria	Savino
Emily R	Schriver
Petra	Schubert
Juergen	Schuettler
Luigia	Scudeller
Neil J	Sebire
Pablo	Serrano-Balazote
Patricia	Serre
Arnaud	Serret-Larmande
Mohsin A	Shah
Zahra	Shakeri Hossein Abad
Domenick	Silvio
Piotr	Sliz
Jiyeon	Son
Charles	Sonday
Andrew M	South
Francesca	Sperotto
Anastasia	Spiridou
Zachary H.	Strasser

Amelia LM	Tan
Bryce W.Q.	Tan
Byorn W.L.	Tan
Suzana E	Tanni
Deanne M	Taylor
Ana I	Terriza-Torres
Valentina	Tibollo
Patric	Tippmann
Emma MS	Toh
Carlo	Torti
Enrico M	Trecarichi
Andrew K	Vallejos
Gael	Varoquaux
Margaret E	Vella
Guillaume	Verdy
Jill-Jênn	Vie
Shyam	Visweswaran
Michele	Vitacca
Kavishwar B	Wagholikar
Lemuel R	Waitman
Xuan	Wang
Demian	Wassermann
Griffin M	Weber
Martin	Wolkewitz
Scott	Wong
Zongqi	Xia
Xin	Xiong
Ye	Ye
Nadir	Yehya
William	Yuan
Joany M	Zachariasse
Janet J	Zahner
Alberto	Zambelli
Harrison G	Zhang
Daniela	Zöller
Valentina	Zuccaro
Chiara	Zucco
